# A clinical trial of enteral Levetiracetam for acute seizures in pediatric cerebral malaria

**DOI:** 10.1186/s12887-019-1766-2

**Published:** 2019-11-01

**Authors:** Gretchen L. Birbeck, Susan T. Herman, Edmund V. Capparelli, Fraction K. Dzinjalamala, Samah G. Abdel Baki, Macpherson Mallewa, Neema M. Toto, Douglas G. Postels, Joseph C. Gardiner, Terrie E. Taylor, Karl B. Seydel

**Affiliations:** 1 Department of Neurology, University of Rochester, 265 Crittenden Blvd, Rochester, NY 14642 USA; 2 Blantyre Malaria Project, Blantyre, Malawi; 3 Department of Neurology, Barrow Neurological Institute, Phoenix, AZ USA; 4 University of California San Diego, Center for Research in Paediatric and Developmental Pharmacology, La Jolla, CA USA; 5 Malawi-Liverpool-Wellcome Trust Research Programme, Blantyre, Malawi; 6 Bio-Signal Group Co., Acton, MA USA; 7 Department of Paediatrics, Queen Elizabeth Central Hospital, Blantyre, Malawi; 8 Department of Neurology, Children’s National Medical Center, Washington, DC USA; 9 Department of Epidemiology & Biostatistics, Michigan State University, East Lansing, MI USA; 10 Department of Osteopathic Medical Specialties, Michigan State University, East Lansing, MI USA

**Keywords:** Acute symptomatic seizures, Tropics

## Abstract

**Background:**

Acute seizures are common in pediatric cerebral malaria (CM), but usual care with phenobarbital risks respiratory suppression. We undertook studies of enteral levetiracetam (eLVT) to evaluate pharmacokinetics (PK), safety and efficacy including an open-label, randomized controlled trial (RCT) comparing eLVT to phenobarbital.

**Methods:**

Children 24–83 months old with CM were enrolled in an eLVT dose-finding study starting with standard dose (40 mg/kg load, then 30 mg/kg Q12 hours) titrated upward until seizure freedom was attained in 75% of subjects.

The RCT that followed randomized children to eLVT vs. phenobarbital for acute seizures and compared the groups on minutes with seizures based upon continuous electroencephalogram. Due to safety concerns, midway through the study children allocated to phenobarbital received the drug only if they continued to have seizures (either clinically or electrographically) after benzodiazepine treatment. Secondary outcomes were treatment failure requiring cross over, coma duration and neurologic sequelae at discharge. PK and safety assessments were also undertaken.

**Results:**

Among 30 comatose CM children, eLVT was rapidly absorbed and well-tolerated. eLVT clearance was lower in patients with higher admission serum creatinine (SCr), but overall PK parameters were similar to prior pediatric PK studies. Within 4 h of the first dose, 90% reached therapeutic levels (> 20 μg/mL) and all were above 6 μg/mL. 7/7 children achieved seizure freedom on the initial eLVT dose.

Comparing 23 eLVT to 21 phenobarbital patients among whom 15/21 received phenobarbital, no differences were seen for minutes with seizure, seizure freedom, coma duration, neurologic sequelae or death, but eLVT was safer (*p* = 0.019). Phenobarbital was discontinued in 3/15 due to respiratory side effects.

**Conclusion:**

Enteral LVT offers an affordable option for seizure control in pediatric CM and is safer than phenobarbital.

**Trial registration:**

NCT01660672.

NCT01982812.

## Background

Cerebral malaria (CM) primarily affects African children [1]. Between 15 and 25% of CM children die and a third of survivors suffer neurologic sequelae [2–4]. Seizures, a common complication, are also a risk factor for post-CM brain injury [2, 5]. CM-associated seizures are prolonged, repetitive, focal, and refractory with subclinical seizures occurring in 18–47% [2, 6, 7]. Seizure management is challenging because phenobarbital and benzodiazepines remain the primary treatments, respiratory suppression is a common complication of both medications, and ventilatory support is generally unavailable [8]. Management of CM-associated seizures in most malarial regions entails some degree of tolerance for ongoing seizure activity due to the risk of respiratory compromise and death with aggressive use of phenobarbital. Whether levetiracetam is effective in this environment for acute symptomatic seizures has been identified by the World Health Organization as a top research priority [9].

Even a short course of parenteral LVT is cost prohibitive in low income settings, but a 3-day supply of LVT oral solution for a 10 kg child is <$15. Pharmacokinetic (PK) studies of enteral LVT (eLVT) in healthy subjects indicate excellent bioavailability with rapid absorption, [10] but aspiration risks in comatose children are unknown. Pharmacokinetics are linear in children from 20 to 60 mg/kg/day. LVT is < 10% protein-bound, 66% of the dose is renally excreted unchanged, and 24% undergoes enzymatic hydrolysis. Metabolites are inactive and renally excreted. LVT clearance is reduced with impaired renal function [10].

We undertook a series of studies to evaluate the safety, PK properties and efficacy of eLVT for acute CM seizure management in children including (1) PK evaluations in a dose-escalation study to identify a potentially effective dose for CM seizures, (2) safety assessments, and (3) an open-label, randomized controlled trial (RCT) comparing eLVT to phenobarbital. Given the ubiquitous nature of subclinical seizures in CM, efficacy was based upon ‘minutes with seizure’ using continuous electroencephalogram (cEEG) monitoring. Duration of coma and neurologic sequelae at discharge were secondary outcomes. Children with seizures, either clinically or electrographically, after the allocated treatment crossed over or received the alternate agent.

## Methods

### Study design

#### Dose-escalation study

A dose-escalation study titrating eLVT to seizure freedom based upon cEEG and limited by toxicity with pre-specified stopping rules.

#### Randomized controlled trial

Using the dose of eLVT identified as effective in the Dose-Escalation Study, we conducted an open label, RCT of eLVT compared to phenobarbital for acute seizure control. PK data were obtained throughout. See study protocol, Additional file 1.

### Patient population

Studies were conducted on the Paediatric Research Ward (PRW) of Queen Elizabeth Central Hospital in Blantyre, Malawi [2, 11, 12]. All aspects of this study received ethical review in Malawi and the US.

#### Inclusion criteria

PRW admission; age 24–83 months; Blantyre Coma Score (BCS) ≤2 [13]; *P. falciparum* infection [14]; no other known coma etiology; seizures within past 24 h; guardian written consent.

#### Exclusion criteria

Serum creatinine (SCr) > 2 mg/dL; use of enzyme-inducing medication in past 14 days; contraindication for nasogastric tube (NGT) and/or administration of enteral medications. For the RCT, additional exclusions were treatment with > 2 doses of short-acting antiseizure drug (ASD) in the past 12 h or a long-acting ASD in the past 3 days.

### Randomization

Patients were block randomized with randomly selected block sizes of 4 and 6. Treatment was allocated based on a pre-defined randomly generated list with assignment available through the OpenClinica database or sequentially numbered, sealed opaque envelopes prepared by the Biomedical Research Informatics Core at Michigan State University. Acquisition of treatment assignment required the enrolling clinician to provide the name and demographic details for the consented subject. Ward clinicians enrolled participants and commenced treatment based upon allocation.

### Procedures

#### Dose-escalation study

Four dose strata of eLVT with a maximum of 8 subjects per strata were prespecified. The initial dose was 40 mg/kg load, then 30 mg/kg Q12 hours for 72 h. Stopping was indicated if mortality plus grade 3 or 4 suspected adverse drug reactions (SADR) exceeded the ward baseline mortality rate of 16%. LVT serum levels were measured at time (t) = 0, 1.5, 4, 12, 24, 36, 40, and 84 h. LVT oral solution (100 mg/ml) was administered via NGT until the child was able to take LVT orally.

#### RCT

Children allocated to eLVT received the optimal dose identified in the Dose-Escalation Study for 72 h. PK data were assessed at t = 0, 7, 24 h and 4–40 h after the last dose. If clinical or electrographic seizures recurred/continued after the initial eLVT dose, an LVT level at time of failure was captured, escalation to the next dosing strata occurred, and the more frequent PK assessments were conducted (t = 0, 1.5, 4, 12, 24, 36, 40, and 84 h). Benzodiazepines (maximum 2 doses/24 h) were given as needed for breakthrough seizures to allow time for eLVT absorption.

The dose escalation study was designed to identify the dose which would be effective for most patients but did not delineate what dose of eLVT might benefit a child with refractory seizures/status epilepticus. Escalating therapy for any selected antiepileptic is the commonest approach to treating refractory seizures. Thus, given the established safety profile of eLVT in other populations, data from other populations indicating that higher doses can be beneficial for refractory seizures, our ability to rapidly cross over to PB if higher dose LVT was ineffective and the value of having additional insights into the clinical and pharmacokinetic effects of escalating eLVT in this population, dose escalation of eLVT was undertaken for those children who failed standard dose.

In RCT year 1, children randomized to ‘usual care’ received phenobarbital 20 mg/kg load, then 5 mg/kg Q12 hours for 72 h. In RCT year 2, the Study Monitoring Committee (SMC) recommended that children randomized to ‘usual care’ receive phenobarbital only if they experienced post enrollment seizures (clinical or electrographic) unresponsive to diazepam or paraldehyde. Children who continued to have seizures after receiving the allocated treatment were given the alternative therapy for rescue.

#### PK studies

High performance liquid chromatography (HPLC) method determined LVT levels [15]. PK data were analyzed using a mixed-effects population approach using the computer program NONMEM (ver. 7.2 Icon, Dublin) and the First Order Conditional Estimation (FOCE) method. Size was incorporated into the model using a standard allometric approach [16]. Due to the limited study sample size, an exploratory analysis of potential covariates was limited to age, liver function tests and SCr. Reduction in the PK model objective function of at least 7.88 (*p* < 0.005) was required for inclusion into the final PK model. A 1000 replicate bootstrap analysis of the final model was performed using Wings v 7.4 to determine the parameter confidence interval. Monte Carlo simulations (15,000 virtual subjects) of the final model and dosing were performed to determine the frequency of achieving trough concentrations 6–20 g/mL.

Since a key concern of eLVT administration in this population was adequate absorption, LVT concentrations collected early in the dose interval (< 4 h after the prior dose) were compared to their (individual) predicted values. The early measured LVT concentrations that were less than 30% of predicted were defined as having slow absorption. Individual subject steady-state trough concentrations, area under the plasma concentration time curve (AUC), apparent clearance (CL/F) and half-life (t_1/2_) were generated using a post hoc empiric Bayesian approach. In this analysis, doses defined as having slow absorption were modelled with an absorption lag time.

#### Electroencephalography

All enrolled subjects underwent cEEG using a *microEEG™* system (Bio-Signal Group Corp), with 21 scalp EEG electrodes placed according to the International 10–20 system. Electrographic seizures were defined based upon standard criteria [17]. Additionally, data was ascertained regarding seizure focality, electrographic seizure duration, the presence and duration of any clinical correlate and the presence and nature of any periodic EEG patterns including lateralized periodic discharges (LPDs) and lateralized rhythmic delta activity (LRDA).

#### Safety assessments

Hematologic, hepatic and renal laboratory assessments were made at baseline, 24 h and 7 days post LVT initiation. An electrocardiogram (ECG) was obtained at baseline and 3 h. See Additional file 2 for Graded Toxicity Criteria. Coma duration was determined and examination at discharge identified neurologic sequelae.

#### Concomitant interventions

All treatments routinely used in the acute care of children with CM as delineated in Malawi National Guidelines and consistent with WHO recommendations were provided [14].

### Outcomes

#### Dose-escalation

The primary outcome was the dose of eLVT for seizure freedom in at least 75% of participants. Secondary outcomes were frequency of vomiting, aspiration, NGT complications, aggression/irritability, coma duration, death and pre-specified adverse events (AE).

#### RCT

Primary outcome was minutes with seizure in the 72 h after treatment allocation based on cEEG data transmitted to interpreter blinded to allocation. Secondary outcomes included efficacy failure requiring cross over, coma duration, neurologic sequelae at discharge based upon a detailed neurologic assessment completed by a clinician who was not blinded to treatment received, death, and safety assessments. ‘Treatment failure’ was defined as any additional seizures, including electrographic, subclinical seizures, after administration of the treatment.

### Statistical analysis

#### Dose escalation study

Up to 8 study participants were to be administered one of four pre-specified doses of LVT. EFFICACY ANALYSIS: Beginning with the initial LVT loading dose (40 mg/kg load, then 30 mg/kg Q12 hours), the target response was seizure freedom in 75% of participants in that stratum for 24 h. We estimated the probability *p* of target response by the proportion of study participants meeting the target response. If the estimate of *p* was less than 0.75, the next higher dose would be used in the next group of children and *p* is re-estimated [18]. Dose escalation would be stopped if the lower limit of the CI exceeded 0.50; otherwise, escalation to the next dose level was indicated. In this scenario, an exact 90% CI for *p* based on 7 responses is (.529, .994) which met the imposed condition. Throughout the dose-escalation study we also monitored for toxicity and acute mortality. Toxicity Analysis: Let *p* denote the event probability of mortality plus grade 3 or 4 SADR in the LVT treatment group. The historic ward case fatality in CM is *P*_0_ (=.16). A non-inferiority test *H*_0_: *p*-*p*_0_ ≥ δ vs H_1_: *p*-*p*_0 <_ δ was carried out where δ (> 0) was the acceptable margin of indifference between LVT and usual care. The conclusion from rejecting H_0_ in favor of H_1_ means that LVT is non-inferior to usual treatment.

#### RCT

Two groups of 30 were planned to receive the LVT dose identified in the Dose-Escalation Study or usual care with phenobarbital (PB). Baseline characteristics between the LVT and PB groups were compared using chi-square tests for categorical variables. For normally distributed continuous variables we applied (independent) t-tests. The validity of normalcy was examined by graphical techniques, using histograms and QQ-plots. Where normalcy was untenable, the log-transformation was applied to mitigate skewness, and if sufficient, t-tests were used. If not, we used the nonparametric Mann-Whitney-Wilcoxon test for independent samples. Additional details are supplied in Additional file 3.

Primary Endpoint: Minutes with seizure during the first 72 h after treatment. Secondary Endpoints*:* (1) treatment failure requiring alternate therapy, (2) time to coma resolution, (3) neurologic sequelae at discharge, (4) acute mortality.

LVT was expected to have a positive effect on outcomes, thus treatment effect was seen in a relative risk *ω* < 1 for an undesirable event such as mortality or presence of neurologic sequelae at discharge, while *ω* > 1 for a desirable event such as seizure freedom for 24 h after treatment initiation. The null hypothesis was H_0_:*ω* = 1 We estimated that with usual care ~ 25% of study participants would be seizure free for 24 h after initiation, whereas with LVT > 60% of study participants would have this outcome, that is *ω* > 2.4. With 30 participants in each arm, the power to detect this difference is over 79% [19]. If approximately 50% of study participants receiving usual care had neurologic sequelae at discharge, whereas with LVT approximately 17% of study participants were affected, that is *ω* ≈ .34, the power to detect this difference is ~ 79%.

#### PK study

In healthy subjects, the %CV is 30% [20]. We used an assumed between subject variability of 50% to account for the expected increased variability in PK that could be encountered in the CM population. Assuming that the between-subject variability for CL/F (clearance/bioavailability) is approximately 50%, with 16–20 subjects the mean CL/F would have a 95% likelihood of being within 25% of the true population mean CL/F value.

## Results

### Dose-escalation study

From February 15–April 15, 2013, 40 children were screened, 11/40 met eligibility criteria and 7 were enrolled. The primary reason for exclusion was no *P. falciparum* infection. Two eligible children were enrolled in another research study whose enrollment alternated with the LVT study and 2/11 were screened when there was no cEEG bed available. The first seven children who received LVT 40 mg/kg load plus 30 mg/kg Q12 were seizure free, so no dose escalation was undertaken. Demographic and clinical characteristics are detailed in Table 1.

**Table 1:** Demographic and Clinical Data from Dose-Escalation Study Population Receiving Enteral Levetiracetam 40 mg/kg load and 30 mg/kg Q12 hourly (*n* = 7)

Characteristic	
Gender (n, % male)	3/7, 43%
Age in months	Mean 54.3; median 53; range 26–81
Retinopathy positive (n, %)	4/7, 57%
Admission hypoglycemia* (n, %)	0/7, 0%
Admission lactate (mmol/L)	Mean 5.4; median 4.5; range 2.2–13.1
Admission hematocrit (% packed cell volume)	Mean 22.2; median 20.7; range 13.0–33.6
Parasitemia (parasite per μl)	Geometric mean 73,700; median 70,000; range 25,920-374,460
Platelets (per μl)	Mean 123,000; median 80,000; range 47,000-259,000
Coma resolution time (hours)	Mean 35.2; median 32.8; range 6.5–78.0

* Glucose < 2.2 mmol/L

The median (range) plasma LVT concentration at 1.5 h was 37.5 (16.7–46.0 μg/mL). Within 4 h of the loading dose, all children achieved at least one LVT concentration between 20 and 50 μg/mL. The median post-load trough was 9.5 μg/mL and after subsequent doses was 7.1 μg/mL. No trough accumulation was noted. No vomiting, aspiration, neurologic sequelae or deaths occurred; 5/7 experienced mildly elevated transaminases and electrolyte perturbations that were already evident at baseline. One child each had QT_C_ prolongation prior to LVT, grade 3 elevation of transaminases and persistent anemia (grade 4)—none of these events were considered related to LVT. All AEs resolved without intervention.

### PK

**Fig. 1:**
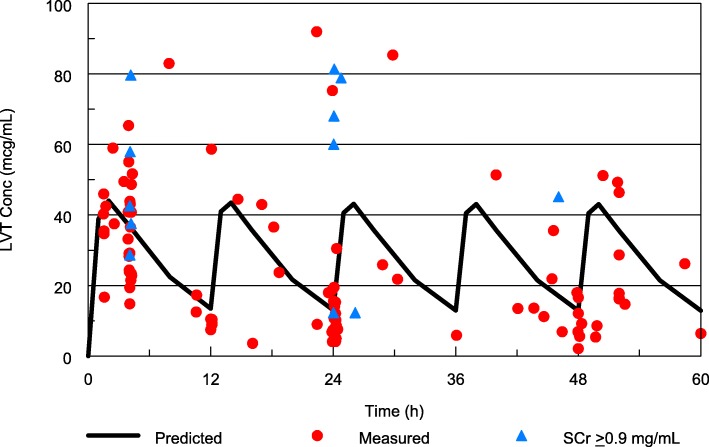
Measured relative to predicted levetiracetam concentrations among children with cerebral malaria receiving enteral LVT stratified by admission serum creatinine. LVT = levetiracetam. SCr = serum creatinine

**Fig. 2:**
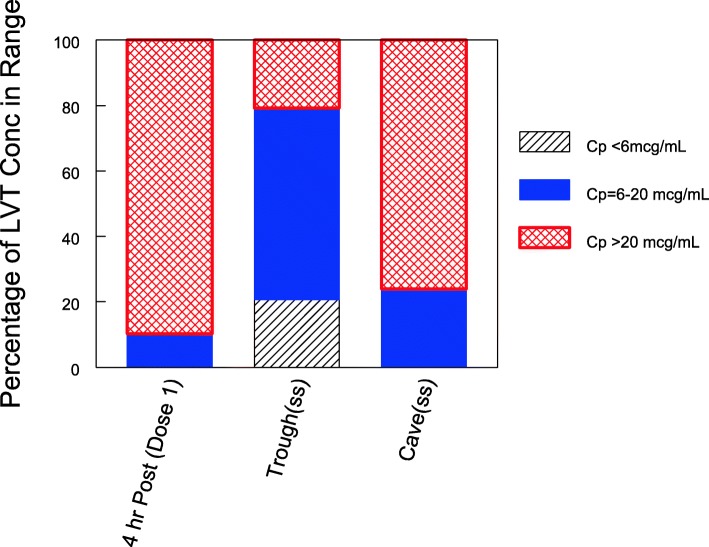
The frequency of observed levetiracetam concentrations 4 h after the first dose and predicted steady-state troughs and average concentrations. All 4 h post first dose and average steady state levels were above 6 μg/mL. LVT = levetiracetam

Therapeutic LVT concentrations were rapidly achieved with LVT levels > 20 μg/mL in 26/29 (90%) within 4 h of the loading dose. No child failed to achieve > 6 μg/mL after the loading dose. The LVT concentrations seen are shown in Fig. 1. Steady-state troughs were below 6 mg/mL in 6/29 (21%) and above 20 μg/mL in 6/29 (21%). Most of the AUC values were between 50 and 200% of the expected population average (Fig. 2). Of the 67 samples collected within 4 h of drug administration, 63 were > 30% of predicted, suggesting relatively normal absorption the vast majority of the time. Only 4 (8%) of the doses administered had altered absorption. In subjects with low initial concentrations, all had additional later samples with adequate concentrations suggesting that delayed rather than incomplete absorption was the issue.

**Fig. 3:**
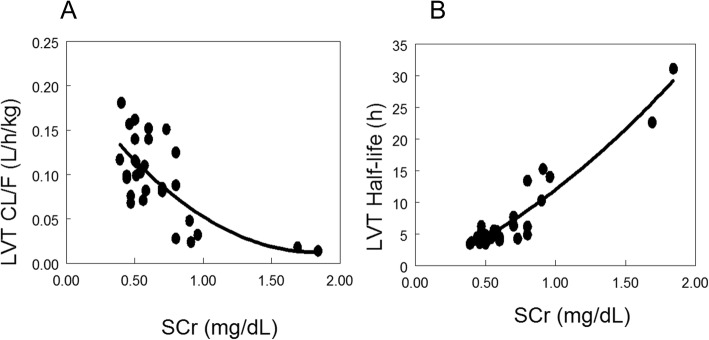
Levetiracetam clearance, levels and half-live in relation to admission serum creatinine. LVT = levetiracetam. SCr = serum creatinine

The population PK analysis utilized 131 LVT concentrations from 30 subjects and was well described by the one-compartment model. Despite the modest number of subjects and samples, CL/F and V/F were estimated with good precision and no bias was evident based on the bootstrap analysis. The estimates for other parameters and between subject variability also were without apparent bias but had lower precision with large standard errors and broad 95% confidence intervals. SCr was found to be a powerful covariate for LVT apparent clearance (objective function reduction of 28.16, *p* < 0.0001), Figs. 3 and 1. Across the range of admission SCr values observed in the study, 0.33 to 1.84 mg/dL, CL/F is predicted to change 10 fold. The final population PK model parameters and precision are summarized in Table 2. The population PK model predicts a typical eLVT apparent clearance (CL/F) of 0.091 L/h/kg for a 3.5 year old weighing 12 kg with a SCr = 0.58.

**Table 2:** Pharmacokinetic Parameters for Enteral Levetiracetam in Children with Cerebral Malaria (*n* = 7)

Parameter	Estimate	SE of Estimate	Median	BS 2.5th	BS 97.5th
V (L/kg)	0.711	0.06	0.696	0.562	0.825
CL (L/h/kg^0.75^)	0.169	0.015	0.171	0.145	0.206
KA (hr^−1^)	1.37	0.286	1.25	0.553	2.37
Lag time (h)	1.96	0.038	1.54	0.258	3.97
SCR factor	−1.37	0.343	− 1.34	−2.06	−0.631
Between Subject Variability					
BSV-V	25%	5%	22%	5%	39%
BSV-CL	43%	6%	42%	28%	56%

V = volume of distribution; CL = total body clearance; KA = absorption rate constant; SCR = serum creatinine

### RCT

**Fig. 4:**
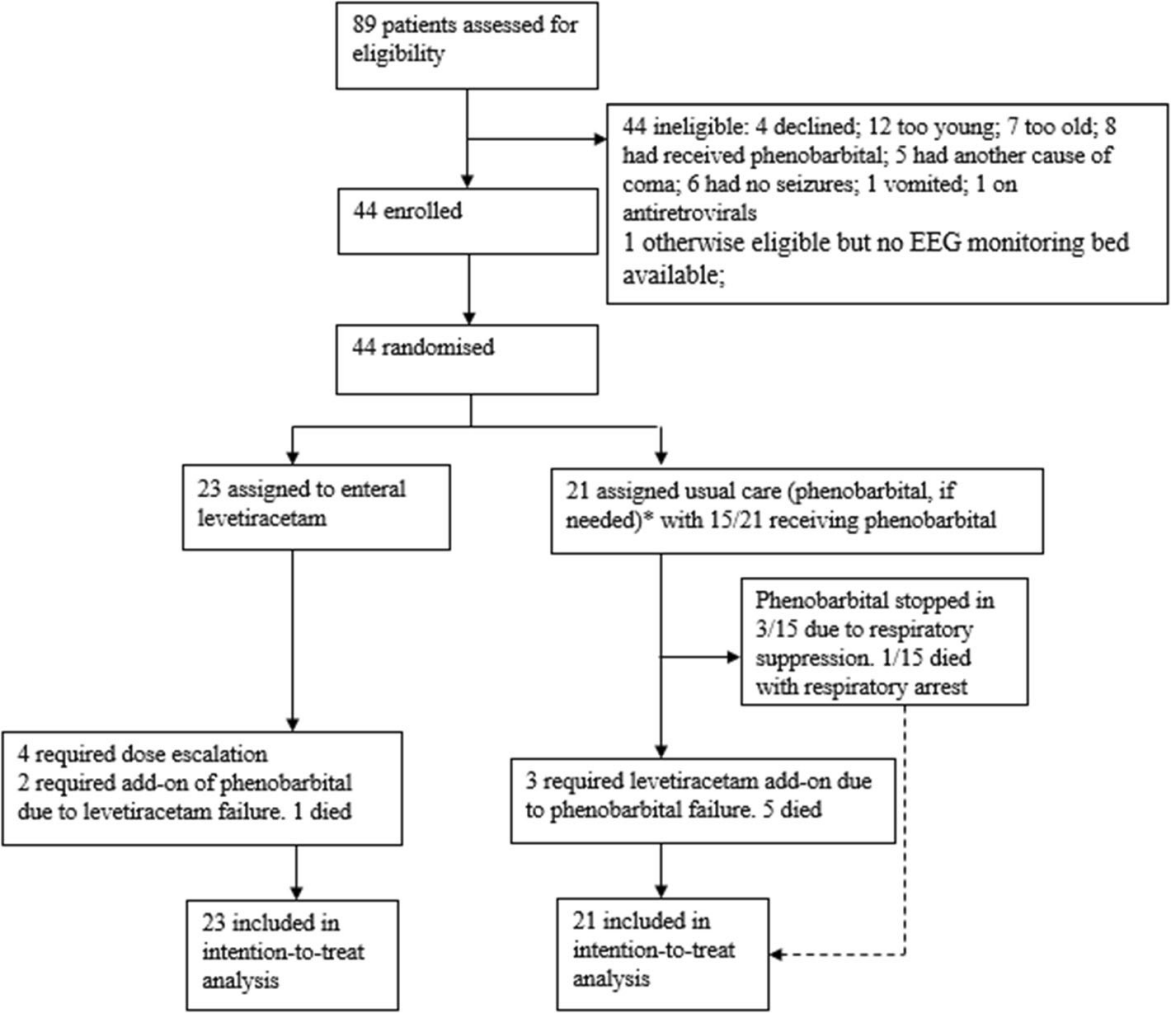
Randomized Control Trial Profile. * “Usual Care” group initially received phenobarbital at enrollment, but protocol revised in 2015 such that “Usual Care” group only received phenobarbital if seizures recurred after allocation

The RCT was conducted January–June in 2014 and 2015. See Fig. 4 for the Trial Profile. Eighty-nine children were screened, 44 enrolled and randomized. The groups did not differ clinically or demographically (Table 3).

**Table 3:** Baseline Characteristics of the Intent-to-Treat Population

	Levetiracetam (*n* = 23)	Usual Care (*n* = 21) (Phenobarbital)	*P*-value*
Age (mean months, SD)	41.4 (10.6)	41.8 (16.7)	0.50
Sex (n, % male)	13 (57%)	7 (33%)	0.14
Parasite count (mean parasite/μL, SD)	214,613 (412,786)	259,729 (314,098)	0.62
Malaria retinopathy (n, % positive)	16 (70%)	12 (57%)	0.35
Lactate (mean mmol/L, SD)	5.4 (3.7)	4.4 (3.3)	0.43
Hemoglobin (mean gdL, SD)	8.5 (3.5)*	8.2 (1.5)	0.41
Hematocrit (% packed cell volumte, SD)	25.8 (11.1)*	24.6 (5.0)	0.63
Platelets (per μL, SD)	163,450 (155,960)α	134,430 (162,330)	0.48
Any rescue benzodiazepine or paraldehyde prior to enrollment¥	8 (35%)	6 (29%)	0.76
Two doses	4 (17%)	2 (10%)	0.66

* Binary variables compared by chi-square tests (exact). Continuous variables compared by t-tests, or non-parametric tests, as appropriate. See Additional file 3 for Evaluation of Normalcy. To mitigate skewness, log-transformation was applied to parasite count, lactate and platelets

α One clotted sample

¥ Diazepam or paraldhyde

All those allocated to LVT received the treatment. Among those allocated to phenobarbital in 2014, 13/13 received phenobarbital per protocol. In 2015, after the protocol was adapted to administer phenobarbital only to children with seizures who failed benzodiazepines, 2/8 received phenobarbital. Overall, in the phenobarbital group, 3/21 required the additional LVT, phenobarbital was stopped in 3/15 due to respiratory suppression and/or aspiration and five died. In the LVT group, 4/23 required dose escalation, 2/23 required phenobarbital and one died.

In the LVT group, 4/23 had breakthrough seizures, mean 165 min duration (SD 266; IQR 26–305; maximum 563). In the usual care group, 5/21 had breakthrough seizures after phenobarbital, mean 465 min (SD 639; IQR 42–734; maximum 1473). There were no differences in minutes with seizure, coma duration, need for alternate treatment, neurologic sequelae at discharge or death. See Table 4. Details regarding neurologic sequelae are provided in Additional file 4. As per our planned analysis, we compared minutes with seizure including all participants. Repeating this analysis but limiting the comparison to those who had any seizure was also not significant (*p* = 0.061). Similarly, the proportion of children with any seizures post enrollment was not different (4/23 vs. 5/21, *p* = 0.072). Despite limiting phenobarbital exposure to those with ongoing seizure in year 2, there was a higher safety failure rate in the usual care group (5/21) compared to LVT (0/23), *p* = 0.019.

**Table 4:** Response to Seizure Treatment and Other Relevant Outcomes

	Levetiracetam (*n* = 23)	Usual Care (n = 21) (Phenobarbital)	*P*-value
Minutes with seizure			*p* = 0.54^
mean (SD) ^~^	165 (266)	465 (639)	
IQR; maximum	26–305; 563	42–734; 1473	
Status epilepticusŦ	2	4	*p* = 0.58
Periodic EEG patterns∞	LPDs (1) GPDs (1)	BIRDS (1) LPDs (4) LRDA (2)	–
Seizure free (n, %)	19 (83%)	16 (76%)	*p* = 0.72 RR 1.08 (95% CI 0.8–1.47)
Required dose escalation	4/23 (17%)	Not applicable	–
Treatment failure, crossed over	2	3	*p* = 0.66
Safety failure^*^	0	5	p = 0.019^#^ RR 0 (95% CI 0–0.59)
Coma durationϯ (mean hours, SD)	*n* = 22 35.4 (29.0)	*n* = 16 34.6 (27.8)	*p* = 0.91
Disposition			*p* = 0.091
-Alive, no sequelae	19	14	
-Sequelae	3	2	
-Died	1	5	

^ Comparison test based upon ranks using a non-parametric test

~ Among only those with seizures

Ŧ Evident both clinically and electrographically in all cases

∞ LPD = lateralized periodic discharges; GPD = generalized periodic discharges; BIRDs = brief ictal rhythmic discharges; LRDA = lateralized rhythmic delta activity

* Drug withdrawal due to SADR. 3/5 respiratory events and 2/5 with concerning decline in coma score after dosing

ϯ Among those who survived

Thirty children received LVT including four escalated to 60 mg/kg load, then 45 mg/kg Q12 hourly higher doses. LVT levels at seizure breakthrough ranged from 36.6–59.0 μg/mL. The LVT level was at least 20 μg/mL in 26/29 (90%) of children within 4 h of the loading dose. The steady state trough was below 6 in 6/29 and above 20 in 6/29 including 4/29 with AUC > 200% of the expected population average.

With one exception, all seizures captured through the continuous EEG monitoring were focal in onset with local propagation. Electrographic generalization was sometimes but not always seen. There was a single generalized onset seizure associated with brief ictal rhythmic discharges (BIRDs) in a PB allocated child.

Clinically evident seizures that appeared generalized in nature evolved from focal electrographically evident subclinical seizures that sometimes waxed and waned for several minutes prior to clinical manifestations being evident. Status epilepticus, meaning seizures lasting longer than 15 min, occurred in 2 LVT allocated children and 4 PB allocated children. In all instances of electrographic status epilepticus, the criteria for clinical status epilepticus were also met but the full extent and duration of ongoing seizures was not evident clinically.

Lateralized periodic discharges occurred in one LVT child and 2 PB children who also had seizures. In addition, 2 PB children who had seizures electrographically had periodic discharges and subsequently died. Generalized periodic discharges were seen in one LVT child. Lateralized rhythmic delta activity was seen in 2 PB children.

AEs are detailed in Additional files 5 and 6. All comparisons for SAEs by allocation had *p* > 0.05 Most were attributed to CM. SADRs attributed to phenobarbital included respiratory suppression, aspiration and prolonged somnolence. Transient myoclonus occurred in one LVT child on awakening and resolved after LVT was stopped.

The Respiratory suppression/aspiration AEs that occurred in four children are detailed below.

- LVT 010: Admission BCs 0/5 but no seizures. Deep coma with shallow respiration and problems handling secretions led clinician caring for the child to elect to stop PB after two doses. After regaining consciousness, the child continued to require oxygen for 24 h and had exam findings consistent with an aspiration pneumonia.
- LVT012: Had received one dose of diazepam prior to randomization. Randomized to PB and within a few hours of admission, after the PB loading dose, developed seizures. No response to paraldehyde so LVT was added. PB was not continued due to physician concerns regarding respiratory suppression once seizures were controlled.
- LVT026: BCS 0/5 on admission. Received PB. EEG with lateralized periodic discharges but no seizures. EEG progressed with slowing and prolonged periods of attenuation for the 24 h after admission, but no respiratory concerns were documented. In the setting of an EEG showing prolonged periods of suppression, an abrupt respiratory arrest occurred and the child died despite bagging.
- LVT033: Admitted with BCS 1, hyperparasitemia, hyperlactatemia and seizures the morning of admission. Randomized to PB with no seizures on cEEG through first 24 h with EEG showing severe slowing on the left and suppression on the right. Due to tenuous respiratory status and decline in BCS to 0, phenobarbital was discontinued at 24 h. EEG continued to worsen with prolonged periods of suppression. Child had respiratory arrest and died at 48 h post enrollment, 24 h after last PB dose.

### Post hoc analysis of LVT elimination with elevated creatinine 

In children with CM, eLVT was well-tolerated and rapidly absorbed. Children with admission SCr ≥ 0.9 had reduced LVT elimination and the highest LVT concentrations. In a post hoc comparison, children with admission SCr ≥ 0.9 had more severe AEs (*p* = 0.0002), all also having at least one grade 4–5 AE compared to 12% of those with SCr < 0.9 (*p* = 0.06) (Table 5).

**Table 5:** Post hoc adverse events in LVT group with admission serum Cr < 0.9 vs. ≥0.9 μg/mL

	LVT Rx, Cr < 0.9 (*n* = 25)	LVT Rx Cr ≥ 0.9 (*n* = 5)	*p* -value	PB Rx, Cr ≥ 0.9 (*n* = 6)
Abnl electrolytes	13 (52)	3 (60)	*p* = 0.57	1 (17)
Abnormal LFTs	11 (44)	3 (60)	*p* = 0.28	3 (50)
Abnl Hematologic	4 (16)	3 (60)	*p* = 0.07	1 (17)
Abnl other	3 (12)	2 (40)	*p* = 0.18	3 (50)
Number of AEs			By category	
0	4 (16)	0		1 (17)
1	10 (40)	1 (20)		2 (34)
2	6 (24)	1 (20)	*p* = 0.17	1 (17)
3	4 (16)	2 (40)		1 (17)
4	1 (4)	0		0
5	0	0		1 (17)
6	0	1 (20)		0
10	0	0		0
Number of AEs	Mean 1.56 (SD 1.04)	Mean 3.80 (SD3.56)	*p* = 0.06	Mean 2.0 (SD 1.8)
AE severity (max)			By category	
0	3 (12)	0		1 (17)
1	10 (40)	0		0
2	6 (24)	0	*p* = 0.001	0
3	3 (12)	0		1 (17)
4	2 (8)	4 (80)		1 (17)
5	1 (4)	1 (20)		2 (34)
Severity of AEs^#^	Mean 1.8 (SD 1.3)	Mean 4.2 (SD 0.45)	*p* = 0.0008	Mean 3.7 (SD 2.0)
Efficacy failure	3 (12)	0	*p* = 0.57	2 (34)

# Signficant at *P*<0.005

## Discussion

This is the first open-label, randomized controlled trial comparing eLVT to usual care with phenobarbital for treatment of acute seizures in resource-limited settings. Among 30 comatose CM children with recent clinical seizures, eLVT was rapidly absorbed and well tolerated. Overall eLVT PK parameters were similar to prior pediatric PK studies, but eLVT clearance was lower in patients with higher admission serum creatinine concentrations. Within 4 h of the first dose, 90% reached therapeutic levels, many reaching therapeutic levels quite rapidly. In the initial dose-finding study, 7/7 children receiving the first planned eLVT dose achieved seizure freedom. In the subsequent randomized comparison of eLVT to usual care patients, no differences were seen for minutes with seizure, seizure freedom, coma duration, neurologic sequelae or death. Although treatment assignment was open-label, the primary and secondary EEG/seizure outcome assessments were masked. eLVT was safer (*p* = 0.019) than phenobarbital, which was discontinued in 3/15 subjects due to respiratory side effects. eLVT therefore is a safe, efficacious, and affordable alternative to usual care for acute symptomatic seizures in this critically ill pediatric CM population who have a high risk of acute seizures, status epilepticus and seizure-associated neurologic sequelae.

Several limitations need to be kept in mind. First, this study provides no insights on eLVT safety in children with SCr > 2 mg/dL. None of the 140 children screened for enrollment had a SCr > 2.0 mg/dL. Caution is warranted in extrapolating the eLVT safety data here to eLVT use for acute symptomatic seizures from causes more often associated with comorbid renal insufficiency. Secondly, we had limited PK data in children with continuous electrographic seizures, [21] but one child who failed standard dose LVT received a higher loading dose while having continuous electrographic seizures and the PK data suggested absorption was delayed until after the seizure halted. Although some of the study subjects developed status epilepticus after enrollment, none of them had experienced status prior to enrollment. The exclusion of children who had received more than two doses of rescue benzodiazepines likely omitted this population from enrollment which may have made the study population less neurologically affected than the typical cerebral malaria population.

Mid-way through the study, the study protocol was amended at the Safety Monitoring Committee’s request based upon interim review of the data which, though not statistically significant at that time, suggested that the risk of respiratory suppression remained elevated in the PB exposed children despite the limitations on pre-enrollment benzodiazepines. This potentially affected the findings in two ways—first, withholding treatment until additional seizures occurred in the PB but not LVT group might have made the LVT treatment appear more efficacious than it would have been compared to PB if everyone in the PB comparison had actually received PB. As such, this study can only confirm that LVT is comparable to PB efficacy when PB is given for ongoing seizures that fail to respond to benzodiazepines. This Safety Monitoring Committee directed protocol change may also have impacted the study findings by making PB appear “safer” than it actually is when given for repetitive but not continuous seizures. This makes the safety findings of this study even more notable.

We also identified some potential issues related to eLVT usage among children with mild renal compromise. Since similar AEs occurred in PB allocated children with elevated admission SCr, modestly elevated admission creatinine is likely an indicator of underlying disease severity rather than high LVT concentrations causing AEs. In terms of efficacy, there was no difference between the LVT and phenobarbital for minutes with seizure, seizure freedom, coma duration, neurologic sequelae or death. The well-established respiratory safety constraints of phenobarbital were evident in this study. The only difference between LVT and usual care with phenobarbital was in the better safety profile of LVT. Given the superior safety profile of eLVT and the option of adding phenobarbital if eLVT proves ineffective, initial management with eLVT is warranted for CM-associated seizures.

## Conclusion

In the setting of acute CM, eLVT is equally effective and has a more favorable side effect profile than intravenous phenobarbital. The availability of a safer ASD in resource-limited settings may result in fewer cases of untreated seizures, as clinicians may be understandably less concerned about inducing respiratory depression in these deeply comatose and critically ill children. Further studies are needed to determine if LVT can decrease neurologic sequelae in CM survivors. Brain injury occurs in ~ 135,000 child survivors of severe malaria in Africa each year. Affordable, effective neuroprotective interventions are certainly needed and would warrant changes in international malaria care guidelines.

## Supplementary information

**Additional file 1.** Study Protocol

**Additional file 2.** Graded toxicity Criteria

**Additional file 3.** Q-Q Plots for Evaluation of Normalcy

**Additional file 4.** Supplementary Table 1: Neurologic Sequelae

**Additional file 5.** Supplementary Table 2: Serious Events by Allocation

**Additional file 6.** Supplementary Table 3: Adverse Events, not Serious

## Data Availability

The datasets used and/or analyzed during the current study are available from the corresponding author on reasonable request.

## Abbreviations

ASD: antiseizure drug
AUC: area under the curve
BCS: Blantyre Coma Score
cEEG: continuous electroencephalogram
CM: cerebral malaria
ECG: electrocardiogram
eLVT: enteral levetiracetam
FOCE: first order conditional estimation
HPLC: high pressure liquid chromatography
IQR: interquartile range
LVT: levetiracetam
NCSE: nonconvulsive status epilepticus
NGT: nasogastric tube
PK: pharmacokinetic
RCT: randomized controlled trial
SADR: suspected adverse drug reaction
SCr: serum creatinine
SD: standard deviation
SMC: study monitoring committee
t: time
t^1/2^: half-life

## References

[1] Idro R, Jenkins NE, Newton CR (2005). Pathogenesis, clinical features, and neurological outcome of cerebral malaria. Lancet Neurol.

[2] Birbeck G, Molyneux M, Kaplan P, Seydel K, Chimalizeni Y, Kawaza K, Taylor T (2010). The Blantyre malaria project epilepsy study (BMPES): neurologic outcomes in a prospective exposure-control study of retinopathy-positive pediatric cerebral malaria survivors. Lancet Neurol.

[3] Carter JA, Mung'ala-Odera V, Neville BG, Murira G, Mturi N, Musumba C, Newton CR (2005). Persistent neurocognitive impairments associated with severe falciparum malaria in Kenyan children. J Neurol Neurosurg Psychiatry.

[4] Roca-Feltrer A, Carneiro I, Armstrong Schellenberg JR (2008). Estimates of the burden of malaria morbidity in Africa in children under the age of 5 years. Tropical Med Int Health.

[5] Newton CR, Warrell DA (1998). Neurological manifestations of falciparum malaria. Ann Neurol.

[6] Crawley J, Smith S, Muthinji P, Marsh K, Kirkham F (2001). Electroencephalographic and clinical features of cerebral malaria. Arch Dis Child.

[7] Gwer S, Thuo N, Idro R, Ndiritu M, Boga M, Newton C, Kirkham F (2012). Changing trends in incidence and aetiology of childhood acute non-traumatic coma over a period of changing malaria transmission in rural coastal Kenya: a retrospective analysis. BMJ Open.

[8] Crawley J, Waruiru C, Mithwani S, Mwangi I, Watkins W, Ouma D, Winstanley P, Peto T, Marsh K (2000). Effect of phenobarbital on seizure frequency and mortality in childhood cerebral malaria: a randomised, controlled intervention study. Lancet.

[9] World Health Organization: Updated guideline: paediatric emergency triage, assessment and treatment. Geneva: World Health Organization; 2016. https://apps.who.int/iris/handle/10665/204463 Accessed 4August2019.

[10] Patsalos PN (2004). Clinical pharmacokinetics of levetiracetam. Clin Pharmacokinet.

[11] Seydel KB, Kampondeni SD, Valim C, Potchen MJ, Milner DA, Muwalo FW, Birbeck GL, Bradley WG, Fox LL, Glover SJ (2015). Brain swelling and death in children with cerebral malaria. N Engl J Med.

[12] Molyneux ME, Taylor TE, Wirima JJ, Borgstein A (1989). Clinical features and prognostic indicators in paediatric cerebral malaria: a study of 131 comatose Malawian children. Q J Med.

[13] Newton CR, Chokwe T, Schellenberg JA, Winstanley PA, Forster D, Peshu N, Kirkham FJ, Marsh K (1997). Coma scales for children with severe falciparum malaria. Trans R Soc Trop Med Hyg.

[14] World Health Organization (2010). Guidelines for the treatment of malaria.

[15] Ratnaraj N, Doheny HC, Patsalos PN (1996). A micromethod for the determination of the new antiepileptic drug levetiracetam (ucb LO59) in serum or plasma by high performance liquid chromatography. Ther Drug Monit.

[16] Holford N, Heo YA, Anderson B (2013). A pharmacokinetic standard for babies and adults. J Pharm Sci.

[17] Hirsch LJ, LaRoche SM, Gaspard N, Gerard E, Svoronos A, Herman ST, Mani R, Arif H, Jette N, Minazad Y (2013). American clinical neurophysiology Society's standardized critical care EEG terminology: 2012 version. J Clin Neurophysiol.

[18] Fleiss J, Levin B, Paik M (2003). Statistical methods for rates and proportions.

[19] SAS/STAT User's Guide (2010). The Power Procedure. In.

[20] Glauser TA, Dulac O (2003). Preliminary efficacy of levetiracetam in children. Epileptic Disord.

[21] Trinka E, Cock H, Hesdorffer D, Rossetti AO, Scheffer IE, Shinnar S, Shorvon S, Lowenstein DH (2015). A definition and classification of status epilepticus--report of the ILAE task force on classification of status Epilepticus. Epilepsia.

