# Supplementary material for: A clinical trial of enteral Levetiracetam for acute seizures in pediatric cerebral malaria
Source: BMC Pediatr. 2019 Nov 1;19:399. doi: 10.1186/s12887-019-1766-2 (PMC6824014; doi:10.1186/s12887-019-1766-2)
Supplement: Supplementary file 1 — Additional file 1. Study Protocol [file 12887_2019_1766_MOESM1_ESM.pdf]

A DOSE-ESCALATION, SAFETY AND FEASIBILITY STUDY OF ENTERAL LEVETIRACETAM FOR SEIZURE CONTROL IN PEDIATRIC CEREBRAL MALARIA

Study Chair:
Gretchen L. Birbeck, MD MPH DTMH FAAN FANA
Professor
University of Rochester, Department of Neurology, Public Health & the Center for Human Experimental Therapeutics
Epilepsy Division, Research Director

Funding Agency:
NIH Exploratory Clinical Trials (R01) PAR-10-199
NIH R01NS074409

TABLE OF CONTENTS

Page

CLINICAL SITES PARTICIPATING IN THE STUDY ……………………………………………4

STUDY TEAM ROSTER ………………………………………………………………………… 5-8

ABBREVIATIONS/ACRONYMS……………….. ……………………………………………….. 9

PRÉCIS ………..…………………………………………………………..…………………10-11

1. STUDY OBJECTIVES ………………………………………………………………..….12

1.1 Primary Objective ………………………………………………………………………12
1.2 Secondary Objectives..……………………………………………..…………….. 12

2. BACKGROUND ………………………………………………………………………..12-16

2.1 Rationale ……....……………………………………………………………………..12-14
2.2 Supporting Data …… ……………………………………..……………………..15-16

3. STUDY DESIGN ……………………………………………………...…………………..16

4. SELECTION AND ENROLLMENT OF SUBJECTS ………………….…..................16-17

4.1 Inclusion Criteria …………………………………………………….……………….16
4.2 Exclusion Criteria ………………………..…………………….………………….....16
4.3 Study Enrollment Procedures ……………………………………………………….16-17
4.4 Randomization Protocol Summary……………………………………………………17

5. STUDY INTERVENTIONS ………………………………………………………………..17-19

5.1 Interventions, Administration, and Duration ...…………………………………………….18
5.2 Handling of Study Interventions …………………………………………………………18
5.3 Concomitant Interventions …………………………………………..………….……….18
5.4 Adherence Assessment …………………………………………………………………..19

6. CLINICAL AND LABORATORY EVALUATIONS ……………………………………………19-26

6.1 Schedule of Evaluations ………………………………………….……………………19
6.2 Timing of Evaluations ……………………………………………..……………………..21-22
6.3 Post-Intervention Evaluations……………… ……………………………………………..22
6.4 Special Instructions and Definitions of Evaluations ………………………………………22-24

7. MANAGEMENT OF ADVERSE EXPERIENCES ……..…………………..………………….24-25

8. CRITERIA FOR INTERVENTION DISCONTINUATION …………...……………………25

9. STATISTICAL CONSIDERATIONS …………………………………………………………..26-32

9.1 Details of Randomization Process…………………………………………………………26

9.2 General Design Issues ……………………………………………….………..................27
9.3 Outcomes ………………………………………………………………..……………………...27-28
9.4 Sample Size, Accrual and Analysis……………………………………………………………28-31
9.5 Data Monitoring …………………………………………………………………...…………..31-32
9.6 Data Analyses …………………………………………………………………………………..32

10. DATA COLLECTION, SITE MONITORING, AND ADVERSE EXPERIENCE REPORTING ……………………………………………………………………………………...32-34

10.1 Records to be Kept …………………………………………………………………………….32
10.2 Role of Data Management ……………………………………………………………………32-33
10.3 Quality Assurance ……………………………………………………………………………...33
10.4 Adverse Experience Reporting ……………………………………………………………....33

11. HUMAN SUBJECTS ……………………………………………………………………………34

11.1 Institutional Review Board (IRB) Review and Informed Consent ……………………..…...34
11.2 Subject Confidentiality ………………………………………………………………………… 34
11.3 Study Modification/Discontinuation …………………………………………….…………….34

12. PUBLICATION OF RESEARCH FINDINGS ……………………………………….…………34

13. REFERENCES ……………………………………………………...………………………35-38

APPENDICES

I. Graded Toxicity Criteria
II. WHO Guidelines for Treatment of Severe Malaria

CLINICAL SITES PARTICIPATING IN THE STUDY (Updated as needed)

Queen Elizabeth Central Hospital
Blantyre Malaria Project (BMP)
Administrator - Esther Gondwe
PO Box 32256
Blantyre 3
MALAWI
Tele: +265-(0)999-969222
Email: egondwe@bmp.medcol.mw

University of Rochester, Department of Neurology
Administrator – Tammie Martin
Box 673
601 Elmwood Ave
Rochester NY 14642
Tele +1-585-275-1791
Fax +1-585-244-2529
Email Tammie_Martin@urmc.rochester.edu

Beth Isreal Deaconess Medical Center
Research Administrator - Allison Dugan
330 Brookline Ave., ST8M-18
Boston, MA 02215-5491
USA
Tele +1-617-667-7328
Fax + 1-617-667-8172
Email: adugan1@bidmc.harvard.edu

UC San Diego, School of Medicine
Administrator – Amy Dittman
Sponsored Projects Office
Department of Pediatrics
9500 Gilman Dr., #0831
La Jolla, CA 92093-0831
USA
Tele +1-858-246-0032
Fax + 1-858-246-0019
Email: adittman@ucsd.edu

STUDY TEAM ROSTER

Principal Investigator
Gretchen L. Birbeck, MD MPH DTMH FAAN FANA
Professor, University of Rochester, Departments of Neurology and Public Health, Center for Human Experimental Therapeutics
Research Director, Epilepsy Division
265 Crittenden Blvd
CU 420694
Rochester, NY 14642-0694
USA
Office +1-517-273-4265
US Cell +1-517-505-0283
SkypeIN +1-517-639-0272
In Malawi +265-(0)995008069
Email Gretchen_Birbeck@URMC.Rochester.edu
SkypeID: muyucottage

Study Coordinator (Malawi)
Ashley Mpakiza, COG
Blantyre Malaria Project
PO Box 32256
Blantyre 3
MALAWI
Cell +265-(0)888855739 +265 111585655
Email ashleympakiza@gmail.com

University of Rochester Study Coordinator
Roseanna Battista
University of Rochester
Center for Human Experimental Therapeutics
265 Crittenden Blvd
CU 420694
Rochester, NY 14642-0694
USA
Office +1-585-275-1642
Cell +1-585-749-8341
Email roseanna.battista@chet.rochester.edu
SkypeID roseanna.battista

MSU Study Coordinator
Doozie Russell
Michigan State University
Department of Neurology & Ophthalmology
Clinical Center
#138 Service Road, A217
East Lansing, MI 48824
USA
Office +1-517-884-2274
Cell +1-517-290-2048
Email: Doozie.Russell@hc.msu.edu
SkypeID LVT.Study

Biostatistican/Clinical Trialist
Joseph C. Gardiner, PhD
Director, Division of Biostatistics
Interim Chair, Department of Epidemiology and Biostatistics
909 Fee Road, West Fee Hall, Room B629
East Lansing, MI 48824-1315
USA
Tele +1-517-353-8623
Fax +1-517-432-1130
East Lansing, MI 48824-1315
USA
Email jgardiner@epi.msu.edu

Bioinformatics Specialists
Nicole Jones-Supervisor
Clinical and Translational Sciences (CTSI)
Biomedical Research and Informatics Center
Michigan State University
909 Fee Road, Room B500
East Lansing, MI 48824-1327
USA
Tele +1-517-432-4149
Fax +1-517-353-9420
Email Nicole.Jones@hc.msu.edu

Khalid Ibrahim
Programmer
Clinical and Translational Sciences (CTSI)
Biomedical Research and Informatics Center
Michigan State University
909 Fee Road, Room B500
East Lansing, MI 48824-1327
USA
Tele +1-517-432-4149
Fax +1-517-353-9420
Kahlid.Ibrahim@hc.msu.edu

Clinical Pharmacologist
Edmund Capparelli
CSD/RCHSD Pediatric Pharmacology Research Unit
7910 Frost Street #360
San Diego, CA 92123-2771
USA
Tele +1-858-246-0001
Fax +1-858-246-0025
Email ecapparelli@ucsd.edu

Fraction Kunseu Dzinjalamala
Malawi-Liverpool-Wellcome Trust Research Program
Box 30096
Blantyre 3

Malawi
Tele +265-(0)999-631295
Email: fkdz72@yahoo.com

Epileptologist

Susan T. Herman, MD
Beth Israel Deaconess Medical Center
Department of Neurology, Baker 5
330 Brookline Ave.
Boston, MA 02215
USA
Tele +1-617-632-8934
Fax +1-617-632-8931
Email: sherman2@bidmc.harvard.edu

Pediatric Neurologists

Macpherson Mallewa
Consultant Paediatric Neurologist/
Senior Lecturer/Deputy Head-Department of Paediatrics
Core Paediatrician-MLW
College of Medicine Malawi-Liverpool-Wellcome Trust Clinical Research Programme
P.O. Box 30096, Chichiri
Blantyre 3
Malawi
Tele +265-1-876444
Tele +265- (0)999-5835083
Email mmallewa@mlw.medcol.mw

Douglas Postels
International Neurologic & Psychiatric Epidemiology Program (INPEP)
Michigan State University
909 Fee Road, West Fee Hall, Room 324
East Lansing, MI 48824
USA
Tele: +1- 517-884-0276
Fax: +1-517-884-0275
Email Douglas.Postels@hc.msu.edu

Malaria Specialists (BMP Clinicians)

Terrie E. Taylor
Osteopathic Medical Specialties
College of Osteopathic Medicine
909 Fee Road, West Fee Hall, Room B309-B
E. Lansing MI 48824-1315
USA
Tele +1-517-353-3211
In Malawi +265-(0)999-969220
Fax +1-517-432-1062
Email taylort@msu.edu

Karl Seydel

Dept of Internal Medicine
College of Osteopathic Medicine
909 Fee Road, West Feel Hall, Room B305-B
East Lansing MI 48824-1315
UTele +1-517-353-3211
In Malawi +265-(0)999-452989
Fax +1-517-432-1062
Email seydel@msu.edu

Study Monitoring Committee Chair

Elizabeth Molyneux
Prof of Paediatrics
College of Medicine
Box 360
Blantyre
Malawi
Tele +265-888-844517
Email emolyneux@malawi.net

Study Monitoring Committee Member

Peter N. Kazembe
Professor of Paediatrics
Lilongwe General Hospital
Lilongwe
Malawi
Tele +265-(0)888-822447
Email pnkazembe@malawi.net

Moffat Nyirendra
Research Scientist
Malawi Liverpool Wellcome Trust Research Programme
Blantyre
Malawi
Tele +265-(0)991-451801
Email mnyirendra@mlw.medcol.mw

Laura Newberry, MD, FAAP, DTM&H
Lecturer of Paediatrics and Child Health
Paediatric Post-Graduate Program Coordinator
College of Medicine
Private bag 360
Blantyre
Malawi
Cell +(265) 997079982
Email lauraanewberry@gmail.com

ABBREVIATIONS AND ACRONYMS

A & E=Accident and Emergency (i.e. Emergency Department)
AE=adverse events
AED=Antiepileptic Drug
ARVs=antiretroviral medications
ATT=antituberculous treatments
BIRB= Michigan state University Biomedical Internal Review Board
BMP=Blantyre Malaria Project
BMPES=Blantyre Malaria Project epilepsy Study
BRIC=Biomedical Research and Informatics Center
CDE=common data elements
cEEG=continuous EEG monitoring
CM=Cerebral malaria
CNS=central nervous system
COMREC=University of Malawi college of Medicine Research Ethics Committee
CRF=Case Report Form
CRM=continual reassessment method
DCC=Data Coordinating Center
eLVT=enteral levetiracetam
EEG=electroencephalogram
ESQ=Epilepsy Screening Questionnaire
FDA=Food and Drug Administration
FWA=Federal Wide Assurance
HIV=human immunodeficiency virus
INPEP=International Neurologic and Psychiatric Epidemiology Program
IV=intravenous
LVT=Levetiracetam
MDAT=Malawi Developmental Assessment Tool
MOP=Manual of Operating Procedures
MRI=Magnetic resonance imaging
MSU=Michigan state University
NESS=Neurologic examination for subtle signs
NGT=nasogastric tube
NIH=National Institute of Health
NINDS=National Institute of Neurological Disorders and Stroke
PCV=plasma cell volume
PI=principal investigator
Pk=pharmacokinetic
QECH=Queen Elizabeth Central Hospital
SADR=suspected adverse drug reaction
SMC=Study Monitoring Committee
WHO= World Health Organization

PRÉCIS

Study Title
A dose-escalation, safety and feasibility study of enteral levetiracetam for seizure control in pediatric cerebral malaria

Primary Objectives
1. To evaluate the safety and feasibility of eLVT use for acute seizure control in pediatric CM
2. To identify the optimal dose of eLVT for seizure control in acute CM by escalating from standard to up to three times standard dose of LVT titrating to the goal of achieving seizure freedom in the 24 hours after LVT administration in 75% of subjects. Seizures will be defined based upon clinical semiology and cEEG. Stopping rules will be imposed for a combined outcome of case fatality and suspected severe adverse drug reaction.
3. To describe the Pk characteristics of eLVT when used in study participants age 24-83 months old with acute CM

Secondary Objectives
1. To obtain preliminary efficacy data on eLVT for seizure control in acute CM by giving 30 study participant optimal dose eLVT and comparing their time with seizure activity to 30 study participants with CM who receive standard AED treatment. Time with seizure will be determined based upon cEEG. The eLVT study participant will receive standard AEDs for any breakthrough seizures.
2. To obtain additional information which may be helpful in planning future clinical trials
    (i) AEDs required for breakthrough seizures (dose and agent)
    (ii) Mean time to coma resolution meaning a return to consciousness with a Blantyre Coma Score≥4
    (iii) Neurologic sequelae evident at discharge

Design and Outcomes
Single center dose escalation study to efficacy limited by toxicity

Secondary study includes an open label, randomized clinical trial comparing eLVT to standard AEDs for seizure control in CM with outcomes that include time with seizures (in minutes) in the 72 hours post LVT administration in study participants given optimal dose eLVT vs. routine AEDs.

Interventions and Duration
Primary
Four dose strata with a maximum of 8 subjects per strata have been prespecified—100% standard, 150, 225 and 300%. The dose will not exceed 300% standard 40 milligrams per kilogram of eLVT followed by 30 milligrams per kilogram (or 100% standard dose) via NGT every 12 hours for 72 hours. cEEG monitoring will be undertaken to ascertain subclinical seizures. Hematologic, hepatic and renal laboratory assessments will be made just prior to LVT administration, 24 hours post LVT and 7 days post LVT administration. An ECG will be obtained at baseline and again 3 hours post LVT administration and the cardiac rhythm assessed. LVT serum levels will be measured at time (hours) = 0, 1.5, 4, 12, 24, 36, 40, and 84. A detailed neurologic exam will be completed at discharge. Dose escalation will proceed until 75% of study subjects are seizure free for the 24 hours after LVT administration or toxicity limits escalation.

Secondary Intervention

Once the optimal dose is determined, we will compare 30 study participants with CM and seizures given optimal dose eLVT to 30 study participants receiving standard AED therapy[1] on time with seizures (in

[1] See appendix 2 for details of standard AED therapy for cerebral malaria seizures in Malawi

minutes) in the 72 hours post LVT administration. The same safety assessments will be undertaken as in the primary intervention.

Sample Size and Population
Children (24-83 months) admitted to the Queen Elizabeth Central Hospital's Pediatric Research Ward with CM (defined as *P. falciparum* parasitemia, a Blantyre Coma Score of ≤2, and no other coma etiology evident) and active seizures on admission or within 24 hours of admission including subclinical seizures identified on routine EEG. Up to 8 study participants will be evaluated at each dose stratum for a maximum of 32 study participants (minimum of 7).

Once the optimal eLVT dose is identified, 30 study participants with CM and seizures will receive optimal dose eLVT and they will be compared to study participants receiving standard AED treatment for time (in minutes) with seizures in the 72 hours after enrollment as determined by cEEG.

1. STUDY OBJECTIVES

1.1 Primary Objective

1.1.1 To evaluate the safety and feasibility of eLVT use for acute seizure control in pediatric CM

1.1.2. To identify the most optimal dose of eLVT for seizure control in acute CM by escalating from standard to up to three times standard dose of LVT titrating to the goal of achieving seizure freedom in the 24 hours after LVT administration in 75% of subjects. Seizures will be defined based upon clinical semiology and cEEG.

1.1.3. To describe the Pk characteristics of LVT when used in study participants age 24-83 months old with acute CM

1.2 Secondary Objectives

1.2.1. To obtain preliminary efficacy data on eLVT for seizure control in acute CM by giving 30 study participants optimal dose eLVT and comparing their time with seizure activity to 30 study participants with CM who receive standard AED treatment. Time with active seizure will be determined based upon cEEG interpretation by off-site epileptologist. The eLVT study participants will receive standard AEDs for any breakthrough seizures based upon real-time, clinical interpretations provided by Drs. Birbeck, Mallewa or Postels.

1.2.2. To obtain additional information which may be helpful in planning future clinical trials

- 1.2.2. a. AEDs required for breakthrough seizures (dose and agent)
- 1.2.2.b. Mean time to coma resolution meaning a return to consciousness with a Blantyre Coma Score of ≥ 4
- 1.2.2.c. Neurologic sequelae evident at discharge

2. BACKGROUND

2.1 Rationale

<u>Prior Clinical Experience & Study Rationale</u>

LVT Use in General Pediatric Populations

Levetiracetam (LVT) is a novel AED in its mechanism as it binds to presynaptic vesicle proteins SV2A in the brain and partially inhibits the high voltage N-type calcium channels(4). It is thought to affect SV2A function only under pathophysiologic conditions and therefore is not thought to affect normal brain physiology(5). It also decreases the effects of negative GABA- and glycine-gated allosteric modulators(6). Some studies have suggested that LVT offers an anti-epileptogenic effect beyond its immediate anti-seizure benefit(7-9). LVT received FDA approval for use in study participant >4 years of age in 1999(10). Chronic LVT therapy in study participant has been found to be well tolerated, safe and effective(11-14). Data regarding the use of intravenous LVT for acute seizures and/or *status epilepticus* is more limited, though numerous retrospective reports suggest such use is common, safe and relatively effective. See Strategy Table 1 for details.

Strategy Table 1: A Review of Findings from Studies of LVT Use for Acute Seizure Control in Study Participants

Population	Seizure Characteristics	Outcomes	Adverse events (AE)
32 study participants in Texas ages 2 months to 18 years (15)	Refractory seizures >50% having failed first-line therapy	All responded to LVT	None
11 study participants in North Carolina ages 2 days to 9 years (16).	*Status epilepticus* having failed at least 2 other AEDs	Controlled seizures in 50%	None
4 study participants from Pennsylvania ages 3 weeks to 19 years(17)	Repetitive seizures/*status epilepticus* .Failed other AEDs.	Seizure termination in 2; Decrease in seizure frequency in 1	None
73 study participants in Colorado ages 1 day to 17.8 years (mean 5.6 years)(18)	Serial seizures (79%), single seizure (12%), and *status epilepticus* (8%). Additional AEDs (1-2 agents) also used.	89% seizure free at 1 hour.	Irritability/aggression<br>GI upset<br>Ataxia<br>Increased hunger
10 study participants in Massachusetts mean age 2.0 +/- 1.2 years(19)	Refractory seizures from an exacerbation of medically intractable epilepsy	5 with ≥50% reduction, 2 seizure free, 1 no change	Seizure worsening (possible AE)
A 10 year old with autoimmune hepatitis(20)	Nonconvulsive *status epilepticus*	Seizure resolution	None
10 study participants median age 5 (range 0.08-14 years) in Pennsylvania (21)	*Status epilepticus* or serial seizures	Temporary termination of seizures (3), termination of nonconvulsive *status (2), stopped seizures (3), reduced seizures (2)*	None.
73 study participants receiving LVT within 30 minutes of a seizure(18)	Most patients received LVT for serial seizures (79%), whereas 12% and 8% manifested a single seizure or *status epilepticus*, respectively	Eighty-nine percent of patients remained seizure-free at 1 hour	behavioral effects<br>ataxia<br>gastrointestinal upset,<br>increased hunger
A prospective feasibility study, LEV was applied as first-line treatment in 38 newborns(22)	Seizures were confirmed by EEG	30 infants were seizure free at the week 1. 27 remained seizure free at 4 weeks.	No severe adverse events were noted
A prospective safety and tolerability study of IV LVT in 30 study participants. Given a single dose (50 mg/kg, maximal dose 2500 mg) over 15 minutes(23)	Study population included study participant with multiple seizure types.	An apparent decrease in seizure frequency across all seizure types was noted.	No serious AEs. Minor reactions included sleepiness, fatigue, and restlessness

LVT pharmacokinetics are characterized by extensive and rapid oral absorption (>95%) with peak plasma concentrations achieved in ~1 hour. LVT has low protein binding (<10%), a volume of distribution that approaches total body water (0.7 L/kg), a half-life of 6 hours in pediatric populations, linear pharmacokinetics, and mixed renal and non-cytochrome P450 mediated hydrolysis to UCB057(24). There are no clinically important pharmacokinetic drug interactions between LVT and enzyme inducing anticonvulsants (diazepam, phenobarbital, carbamazepine and phenytoin)(25). LVT is mainly renally cleared and excreted unchanged. The portion of LVT that is metabolized is through hydrolysis indicating that oxidative CYP 450 and specifically CYP 2D6 is not involved. LVT is not a substrate for MDR1 either. Therefore, drug interactions with high dose quinine, antipyretics, lumefantrine, artemether or, dihydroartemisinin, are unlikely making LVT a good candidate for seizure control in CM. Apparent clearance (CL/F) in pediatric study participants is greater than adults although neonates have reduced elimination due to immature renal function(1, 13)(Capparelli – unpublished). Doses of 1000 to 3000 mg/day in adults and 20-60mg/kg/day in study participants are generally well tolerated and achieve target trough concentrations between 6-20 mcg/mL(25, 26).

LVT in other pediatric populations has been found to be extremely safe. Monitoring of laboratory values with LVT use is generally not required since studies to date have indicated there are no meaningful

changes in liver function tests or other blood chemistries(27, 28). Hypersensitivity reactions to LVT in clinical trials are similar to rates reported for placebo(27). The most common side effect reported in studies of LVT use in study participant is behavioral events with hostility, emotional lability, and agitation which resolve with discontinuation of the medication.

LVT Use for Seizure Management in Pediatric CM

Data on the use of LVT in resource limited settings is almost nonexistent. LVT has been used in a private hospital in India, where 30/41 patients (all >14 years old; mean age 21 +/- 10 years) with refractory *status epilepticus* had their seizures controlled with LVT(29). In this study it was specifically noted that recipients did not require ICU-level care. There is presently an ongoing transnational Phase II clinical trial of intravenous IV LVT for *status epilepticus*(30). But LVT is not currently available in the public sector in Africa, largely due to the high cost of the medication. Even generic oral formulations, though potentially affordable for short term use, would be ~$250 per month for an adult--more than 10 times the cost of the most expensive first line agent recommended by the WHO(31). IV LVT is equally unaffordable--$116 for a 2000 mg dose. While the cost of IV LVT is unaffordable for acute seizure management in the public sector of resource limited settings, oral LVT solution at $8.80/2000mg offers a feasible alternative. Enteral delivery of LVT via nasogastric (NGT) offers the additional advantage of allowing primary care facilities in malaria endemic regions to provide treatment when IV access cannot be obtained or maintained. So although there is essentially no efficacy data on the use of LVT for CM, or in fact for any pediatric condition in a resource limited setting, short term use of enteral LVT for acute seizure control could potentially be feasible and affordable.

In addition to issues of efficacy of LVT, important consideration is needed regarding dose selection. CM-related seizures are complex, multifocal, and prolonged with *status epilepticus* being common despite maximally tolerated doses of standard AEDs. See Strategy Figure 1 showing quantitative EEG analysis from a Malawian child with CM receiving standard AED treatment (diazepam, phenobarbital and phenytoin). Given the seizure severity often seen in CM, higher than standard therapeutic doses of LVT may be required for optimal management. Severe malaria may alter drug absorption, distribution and elimination(2). In addition to these systemic metabolic effects of pediatric CM, gut endothelium has been identified as a site of parasite sequestration. Vomiting prior to coma onset in CM is common, possibly due to intussusception which is often seen at autopsy(3). Enteral drug delivery in the first 24-48 hours after admission with pediatric CM is not part of routine practice. To plan a phase III clinical trial of enteral LVT for acute seizure management in African study participant with CM, data are needed regarding the feasibility, safety, pharmaco- kinetic, and preliminary efficacy of this intervention. We will conduct a dose-escalation safety and feasibility study of enteral LVT in pediatric CM using a modified continual reassessment method that incorporates safety, efficacy and pharmacokinetic data to identify an optimal dose. Given the prevalence of subclinical seizures in pediatric CM and critically ill study participant in general(21, 32), efficacy will be defined by seizure freedom based upon continuous EEG monitoring (cEEG). Preliminary data comparing seizure control with optimal dose LVT to standard AED treatments will also be obtained. Capacity building activities include expanding the present capabilities of a relatively new high pressure liquid chromatography (HPLC)

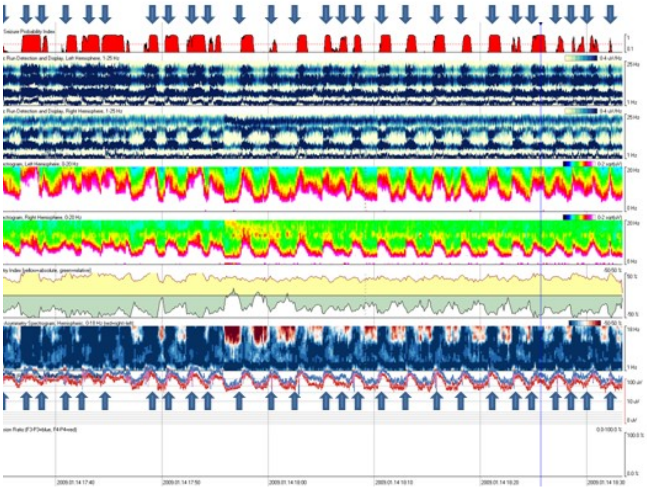

Figure 1:
This quantitative EEG display shows 28 discrete seizures (arrows) in a 1 hour segment of EEG. Seizures are best demonstrated by neural network seizure detection algorithm (Persyst Reveal Rosetta, top arrows), amplitude integrated EEG (bottom arrows), and color spectrogram.

laboratory at QECH to include LVT measurement and incorporating cEEG into care where routine daily EEGs were previously standard of care.

2.2 Supporting Data

Cerebral malaria (CM) is estimated to affect more than 3 million study participant each year resulting in ~ 1 million deaths with 90% of CM-related deaths occurring in Africa(31). The criteria for CM include: [1] coma with no localizing response to pain that persists for more than one hour if the patient has experienced a generalized seizure; [2] asexual forms of *P. falciparum* found in the blood; and [3] exclusion of other causes of encephalopathy(33). Newer antimalarial agents that rapidly clear peripheral parasitemia improve survival but mortality rates remain 12-25%(34-36). Established risk factors for death in pediatric CM include seizures, profound coma, signs of decerebration, absence of corneal reflexes and age under three years(37-43). Laboratory findings associated with a poor prognosis include hypoglycemia, leucocytosis, hyperparasitaemia, elevated plasma concentrations of alanine and 5'-nucleotidase, and elevated plasma or cerebrospinal fluid lactate(44).

CM survivors do not escape unscathed. Past studies have identified neurologic deficits at discharge in 9-18% of survivors with additional sequelae, such as epilepsy, becoming evident with longer term follow-up(43, 45-50). Hearing impairment has also been reported post CM(50). In 2004, an autopsy study of CM in Malawi revealed that the traditional clinical criteria for CM (parasitemia, deep coma and no other evident coma etiology) is nonspecific with ~23% of cases lacking pathological evidence of CNS parasite sequestration and, in fact, having another cause of coma and death identified(51). This diagnostic limitation can be overcome by inclusion of a funduscopic examination. A CM retinopathy originally identified by researchers in Blantyre, Malawi has been found to offer 100% specificity and 95% sensitivity for identification of true CM prior to autopsy(51-56). We conducted a 5-year prospective cohort study of pediatric CM survivors (The Blantyre Malaria Project Epilepsy Study, or BMPES) who met the more stringent diagnostic criteria that include CM retinopathy. In this retinopathy-confirmed CM population of 132 study participant, ~10% had neurologic deficits evident at discharge. When followed for an average of 77 weeks, 32.1% experienced neurologic sequelae including epilepsy (9.1%), behavioral problems (10.6%) and new neurodisabilities characterized by gross motor, sensory, or language deficits (23.1%)(32).

In virtually all studies of CM aimed at identifying adverse neurologic outcomes, including the BMPES study, seizures during the acute infection have been identified as a primary risk factor for adverse outcomes(42, 45, 47-49, 57, 58), with prolonged and/or recurrent seizures being especially common and strongly associated with long-term disabilities and epilepsy. Among CM survivors in BMPES, seizures during the acute admission were strongly associated with later epilepsy (91.7 vs. 63.3%; OR 6.37). Additional risk factors identified for adverse neurologic outcomes include hypoglycemia(45), prolonged coma(45, 47, 48), deeper coma(47, 48, 57), and a higher maximum temperature(57).

CM-associated seizures present a significant challenge in clinical management. *Status epilepticus* and recurrent seizures are common(42, 59). Seizures are frequently focal, though secondary generalization often occurs. Clinical seizure semiology suggests multifocal seizures indicative of more than one irritative structural lesion and this had been confirmed in the few studies where EEG technology has been available to further characterize CM-related seizures(59, 60). The epilepsy described after CM is usually localization-related(61, 62). Among study participant in the BMPES study who developed epilepsy, neuroimaging during follow-up identified focal cortical atrophy that correlated to the region of active, recurrent focal seizures identified by EEG during the acute CM infection and which also correlated to the foci of the patient's localization related epilepsy and other post-CM focal neurologic deficits. Although causality remains uncertain, there was a clear link seen between acute CM-associated seizures, subsequent epilepsy and identifiable structural atrophy among CM survivors in the BMPES study.

Aggressive seizure management in pediatric CM is difficult. Subclinical CM-related seizures without any clinical correlate are common, but EEG technology is rarely available in malaria endemic regions. Subtle seizures were seen in 23% of study participant in Kilifi, Kenya(59). In the BMPES study, among study participant with no suggestion of seizure activity by caregiver history or physician assessment, 19% had active seizures on EEG(32, 60). Unfortunately, the antiepileptic drugs (AEDs) routinely used for acute seizure management in malaria endemic regions include older agents such as diazepam and phenobarbital.

These can all worsen the respiratory suppression that may already be present in deeply comatose study participant and mechanical ventilation is not available even in tertiary care settings. A clinical trial of phenobarbital to determine if more aggressive seizure management could improve survival in pediatric CM had to be halted early due to higher mortality rates in the treatment group—and the deaths were primarily due to respiratory failure(11). Essentially, efforts to control acute CM-related seizures are limited by our inability to identify subclinical seizures as well as the reality that aggressive treatment with available AEDs results in respiratory failure and death.

We hypothesize that more optimal seizure control during acute CM will result in lower rates of adverse neurologic outcomes in survivors. The ideal AED for use in CM would be an AED that is affordable for short term use, feasible to administer in resource limited settings, unlikely to interact with other medications needed to treat CM, unlikely to cause respiratory suppression and is otherwise safe enough to be used in all study participants presenting with clinically diagnosed CM, even those who have no obvious evidence of seizure.

3. STUDY DESIGN

3.1 Dose-escalation titrated to efficacy and limited by toxicity (a combined endpoint of death and severe suspected adverse drug reaction)

3.2 Open label, randomized clinical trial

4. SELECTION AND ENROLLMENT OF SUBJECTS

4.1 Inclusion Criteria

- Admission to QECH Pediatric Research Ward
- Aged 24-83 months
- Comatose with BCS ≤2(40)
- *P. falciparum* parasitemia as detected by thick peripheral blood smear using Field's staining technique(63)
- No other coma etiology evident based upon examination, history and lumbar puncture (where lumbar puncture is deemed safe)
- Active seizures within 24 hours of admission, defined as clinically evident seizures or seizures evident on routine EEG as determined by the interpreting clinician
- Willingness of legal guardian to give written informed consent

4.2 Exclusion Criteria

- Serum creatinine on admission of > 2 mg/dL (using a bedside Cr meter)
- Recent (within 14 days) use of any enzyme-inducing medication including but not limited to current rifampin containing tuberculosis therapy or antiretroviral therapy for HIV infection (including protease inhibitors, nucleoside reverse transcriptase inhibitors, non-nucleoside reverse transcriptase inhibitors, integrase inhibitors or entry inhibitors).
- Contraindication for placement of nasogastric tube and/or enteral medication administration

Additional exclusion criteria for years 2 and 3 when evaluating Aim 2-

- Treatment within the past 12 hours with more than two doses of acute rescue treatment with a short-acting antiepileptic agent (Meaning > 0.4mg/kg IV diazepam; > 0.8 mg/kg PR diazepam; > 0.4 mg/kg IM paraldehyde)
- Treatment within the past 3 days with any long-acting anticonvulsant including phenobarbitone, phenytoin, or carbamazepine

4.3 Study Enrollment Procedures

Mechanisms are already in place to facilitate rapid referral of CM patients to the Pediatric Research Ward. These include active BMP staff stationed in A & E as well as on the Pediatrics Ward. Surveillance of the existing inpatients is critical to identify study participants who develop coma after admission. The Pediatric Research Ward serves as the *de facto* Pediatric Intensive Care Unit for QECH and consent to participate in ongoing research is not required for transfer or admission to the ward (i.e. children are transferred for medical need regardless).

After admission/transfer to the ward and after the child has been stabilized (including treatment of acute seizures), the ward physician will review the admission for eligibility (including completion of a bedside creatinine) and, if the child meets all other inclusion criteria, a trained nurse-counselor will approach the parent/guardian to discuss informed consent for inclusion in this study. If a child admitted without known seizure experiences a seizure in the first 24 hours after admission, either clinically evident or on routine EEG, the ward physician will again review eligibility for the study. Nurse-counselors are native Malawians fluent in the local language of Chichewa. Nurses are not compensated based upon recruitment, but are full-time healthcare workers on the Research Ward where study participants and children admitted for nursing care are not treated differently except for protocol-specific requirements. A complete record of the consent process will be documented in the screening and consent log. A unique identification number will be assigned to study participants in this study. The children admitted to the QECH Pediatric Research Ward are typical of those who suffer from malaria globally each year and are a sub-sample from the greater population that could benefit from neuroprotective interventions if they prove to be effective.

Given that funduscopic examination is not generally available in routine care settings in malaria-endemic regions, it is likely that any potentially efficacious treatments identified for CM would be delivered to the broader group of pediatric CM children which include children meeting the traditional case definition of CM but who lack CM retinopathy and likely have another underlying cause for coma. Therefore, children meeting the WHO case definition for CM who lack retinopathy will also be eligible for this LVT dose finding study to determine overall safety.

For the open-label, randomized clinical trial comparing eLVT to standard AEDs, enrollment will be randomized eLVT or routine care. No more than 3 study participants will be enrolled in the eLVT arm at any time due to equipment limitations and to the burden of assessment for staff in study participant given collection of Pk data.

Due to the need to randomize patients for Aim 2 (the open-label, randomized efficacy assessment) prior to the receipt of any long-acting AED for years 2 and 3 we will also recruit directly from the Special Care Ward and A&E.

4.4 Randomization protocol summary

Patients will be assigned to treatment groups using blocked randomization with randomly selected block sizes (76). After consent, subjects will be assigned to treatment groups based on a pre-defined randomly generated list. Clinical staff will log into OpenClinica to determine subject assignment. For periods of time when OpenClinica is unavailable due to local technical issues, identical assignments will be available in sequentially numbered sealed opaque envelopes for clinic staff use. The two systems will be linked through a paper log.

5. STUDY INTERVENTIONS

Interventions, Administration, and Duration (Dose Escalation Study)

After the parent/guardian consents, a pediatric sized NGT will be placed by experienced staff. The assigned dose of eLVT will be administered. For the study participant receiving the initial dose, this will be a standard 40mg/kg loading dose followed by 30mg/kg Q12 hours (~6 ml for a study participant of 15kg) for 72 hours or a total of 7 doses including the loading dose. For subsequent assigned doses, Pk data from the 100% standard dose cohort will be used for the doses of ~150, 225, and 300% standard. cEEG monitoring will be commenced as the eLVT is being administered and cEEG monitoring will continue until the study participant regains consciousness (reaches BCS ≥4) or dies.

5.1 Interventions, Administration, and Duration (Open Label, Randomized Trial)

After the optimal dose is determined, additional study participants will receive the optimal dose to reach a total of 30 study participants. The comparison group will include study participants who otherwise meet all inclusion criteria but these study participants will receive standard AED treatment[2] only. The only additional monitoring they will undergo beyond that routinely provided on the Pediatric Research Ward will be cEEG rather than the usual daily EEG provided and the safety labs at baseline, 24 hours after treatment is initiated and at follow-up. To standardize the observation period for seizure recurrence, both the LVT and standard treatment groups will have cEEG applied with t=0 for cEEG monitoring to begin no later than 2 hours after consent is given. (This should roughly approximate to 1 hour post eLVT delivery).

For children assigned to the eLVT treatment arm who experience seizures on standard dose eLVT, we will escalate to 150% standard dose and conduct pharmacokinetic assessments in children receiving this higher dose only. The Pk sampling protocol used will be the same as that used in Aim 1, the dose-escalation phase. If seizures continue on 150% standard dose eLVT, the child will the cross over to receive standard AED treatment for rescue therapy. Children assigned to the standard AED treatment arm who continue to have seizures despite maximum therapy will receive standard dose eLVT for rescue therapy with escalation to 150% eLVT if needed. Pharmacokinetic data following the protocol used for Aim one will be obtained.

*Children randomized to PB will receive PB only if they experience further seizures (either clinically or on cEEG) which fail to respond to two doses of rescue treatment with benzodiazepines and/or paraldehyde.*Children who require PB will receive 6 doses after the load to remain comparable to LVT in terms of treatment duration. Once the child is awake and able to swallow, PB may be given orally.

5.2 Handling of Study Interventions

Oral Keppra® solution will be purchased and shipped via courier to Blantyre. Shippers will be provided with details regarding environmental controls which include keeping the medication in moderate temperatures (at 15-30°C) and the necessary measure taken to assure this occurs. In Blantyre, the medication will be stored in the Pediatric Research Ward Pharmacy, located adjacent to the Ward where it will be kept in a medication locker out of direct light. The room will be maintained a between 15-30° Celsius with a dedicated air conditioning unit that is connected to the BMP generator in case of power outages. Supplies will be ordered in the 6 months preceding each recruitment period and shelf-life for unopened contained is 3 years, so substantial left over stocks of medication are not expected.

5.3 Concomitant Interventions

All treatments routinely used in the acute care of children with CM will be provided (63). These include antimalarials (intravenous quinine or an artesunate followed by a full oral course of arteme-

ther-lumefantrine), antipyretics (acetaminophen, ibuprofen), acute seizure treatment as needed (diazepam), other anticonvulsants as needed (phenobarbital followed by phenytoin), and blood transfusions. BMP study procedures include clinical observations (vital signs and BCS) every 2 hours; blood samples for malaria parasite counts, plasma cell volume, glucose, and lactate every 6 hours; and timely blood transfusions when indicated. Lumbar punctures are completed on admission unless contraindicated. The duration and characteristics of all clinical seizures are documented in a "fitting chart" by nurses, clinical officers[3] and physicians on the ward. Seizures on the BMP are treated promptly based upon clinical observations. In addition to treating clinically evident seizures, clinicians can ask for acute review of the cEEG for any clinically suspicious event. Quantitative EEG analysis will be provided to the clinicians caring for all patients undergoing cEEG monitoring just prior to the morning and evening ward rounds to further direct care. When electrographic seizures are identified by the physicians reviewing cEEG on site for clinical purposes and to direct dose escalation or cross over treatment to unassigned drug if needed. The responsible clinicians will be notified immediately so treatment can be initiated or changed as indicated. Study participants enrolled in this study including those receiving eLVT will receive standard treatment for seizure activity (clinical seizure as well as electrographic seizures with no clinical correlate). For study participant who are experiencing a seizure at the time of enrollment, an initial dose of diazepam (our standard first line treatment) will be given and subsequent doses delivered as needed per Ward protocols, which are consistent with WHO guidelines. If treatment is given for cEEG findings interpreted locally as a seizure but Dr. Herman determined the findings was not a seizure, this will be a protocol violation requiring subject replacement. Details for standard ward protocols for seizure management are provided in the Appendix.

5.4 Adherence Assessment

Study drug administration will be undertaken by a Pediatric Research Ward nurse. If/when the study participant awakens, NGTs will be removed and the medication (which is grape flavored) will be given orally. The administering ward nurse will directly observe the oral intake of the medication and document oral adherence. If vomiting occurs with 30 minutes of oral administration, a repeat dose will be given and documentation of the event made.

6. CLINICAL AND LABORATORY EVALUATIONS

[3] Clinical officers are healthcare workers who receive at least three years of training after secondary school

6.1 Schedule of Evaluations

Table 1: Schedule of Evaluations

Evaluation	Pre-Entry	Entry	Day 1	Day 2	Day 3	Day 4	Discharge (d/c)	7 days post eLVT +/-2 days
Informed Consent	X							
Documentation of Disease/Disorder	X					X		
Creatinine measurement (bedside)	X							
Medical/Treatment History	X							
Detailed Neurologic Assessment	X						X	
Physical Exam	X		Twice daily	Twice daily	Twice daily	Twice daily		
Vital Signs including oxygen saturation		X	Q2 hourly until BCS 4, then Q 6 hourly					X
ECG (12 lead)		X	3 hours after eLVT administration, of applicable					
Hematology (CBC) + retic count		X		X				X
Chemistry<br>(electrolytes + renal function)		X		X				X
Liver Function Tests		X		X				X
Thick blood smear (MPs) and lactate	X		Q 6 hourly until negative/normal times 2					
Glucose		X	Q6 hourly for the first 24 hours after admission. More frequently if clinically indicated and as clinically indicated after 24 hours					
LVT Pk Detailed		Before dose 1 (t=0 hours), then at 1.5 hours, 4 hours, 12 hr (prior to 2nd dose) , 24 hr (prior to 3rd dose), 36 hour (prior to 4th dose), 48 hour, and 84 hour						
LVT Pk Limited		Before dose 1, (t=0), at 4 hours post dose 1 and 24 hours (prior to 3rd dose). An additional level will be drawn after dose # 6 between 4 and 40 hours after the last LVT dose.						
cEEG		From eLVT administration until 24 hours post admin for dose-escalation; 72 hours for efficacy pilot study						
Cardiac rhythm, assessment		Via cEEG during administration of loading dose						
Adherence Assessments			Per directly observed therapy if/when agent administered orally					

Note that in Aim 2, the open-label randomized comparison of eLVT to standard treatment with phenobarbitone, only the "LVT Pk limited" studies for Pk will be undertaken. "LVT Pk Detailed" was obtained in year 1 for the dose escalation phase and will also be obtained in study subjects who fail standard dose LVT and escalate to 150% standard or those who receive LVT after failing treatment with standard therapy in Aim 2.

6.2 Timing of Evaluations

6.2.1 Pre-Enrollment Evaluations

These evaluations occur prior to the subject receiving any study interventions.

Screening

Children admitted to the QECH Pediatric Research Ward will be reviewed for inclusion. General admission procedures include thick blood smear for malaria parasites and a clinical assessment which includes the BCS and seizure assessment. Additionally, a rapid creatinine check, and review of exclusion criteria (concomitant use of ATT or ARVs or other enzyme-inducing agent) will be undertaken.

Entry

Eligible children with guardians willing to provide written informed consent will be enrolled. An NGT will be placed and the loading dose of the study drug administered within 4 hours of obtaining consent. cEEG will be applied concurrently—this takes ~35 minutes

6.2.2 On-Study Evaluations

Dosing and serum level evaluations must be tightly adhered to—actual times of each dose and drug level will be carefully documented in the file. The discharge neurologic assessment may occur anytime on the actual day of the discharge from hospital.

6.2.3 Intervention Discontinuation Evaluations

The intervention will be discontinued at the explicit request of the caregiver/guardian. The intervention will also be discontinued if based upon the BMP clinician's judgment LVT-associated adverse effects including vomiting, aspiration or complications related to administration of the study drug warrant discontinuation. A discontinuation form to be included in the CRF, will be completed by the attending physician, must be documented and filed for guardian-initiated withdrawals or discontinuation based upon AEs or elevated concerns regarding for potential AEs. Study participants who discontinue the intervention will remain on the Pediatric Research Ward for the high level care available on this unit.

We will characterize laboratory adverse events according to graded criteria per a modified version of the NINDS Common Data Elements for Adverse Events. Even though LVT has no clear safety signal based on laboratory abnormalities, this is a novel population for LVT use and laboratory assessment is required. All suspected adverse drug reactions (SADRs) will be recorded on CRFs. The core team will be notified of any Grade 3 SADR (including death) within 24 hours of the site being aware of the event along with the PI and clinician's plan for clinical management and follow up of these events as appropriate. Note that since children with CM are expected to have several abnormal laboratory parameters due to their underlying condition, a significant change from admission laboratory values will be the parameter assessed to identify laboratory-based SADRs. These are detailed in the Appendix.

Grade 1 Toxicity: Continue LVT.

Grade 2 Toxicity: Continue LVT.

Grade 3 Toxicity: LVT should be held at the discretion of the BMP clinical director. For abnormal laboratory results, repeat assessment/confirmation should be done as soon as possible. If repeat assessment confirms Grade 3 toxicity, continue to hold LVT and follow abnormal laboratory values weekly. All toxicities should continue to be monitored weekly until the toxicity is Grade 2.

Grade 4 Toxicity: LVT must be held if the adverse event is determined to be possibly related to LVT. For abnormal laboratory results, repeat assessment/confirmation should be done as soon as possible. If repeat assessment confirms Grade 4 toxicity, LVT should be permanently discontinued. If repeat assessment shows Grade 3 toxicity, continue to hold LVT and follow abnormal laboratory values. If toxicity resolves to ≤ Grade 2, LVT can be restarted. If > Grade 3 toxicity recurs after reintroduction of LVT, LVT must be permanently discontinued. All toxicities should continue to be monitored at least weekly until the toxicity is Grade 2 after which the subject will be removed from study.

6.3 Post-Intervention Evaluations

A neurologic assessment will take place on the day of discharge. cEEG will continue beyond 72 hours if the study participant remains with a BCS <4.Laboratory safety assessments made at 7 days post eLVT administration include hematologic, renal and hepatic evaluations. Laboratory abnormalities will be characterized using graded criteria based upon the modified NINDS Common Data Elements for Adverse Events (see Appendix). A detailed neurologic evaluation will also be completed at the 7 day (+/- 2 days) post LVT initiation.

6.3.1 Final Evaluations

The assessment at 7 days (+/- 2 days) post LVT initiation will be the final visit. At that time, study participants will be referred for any further services or care needed based upon the findings in the evaluation.

6.4 Special Instructions and Definitions of Evaluations

6.4.1 Informed Consent

A detailed discussion between the guardian/caregiver and the research nurse seeking consent will take place in a quiet location in Chichewa (the local language). All consent documents and procedures will be approved via COMREC and BIRB. COMREC's FWA number is **FWA00011868** and BIRB's is **FWA00004556.** All the study key personnel have undergone the required training in human subjects' protection and this certification will be maintained throughout the study period. Any and all changes to the consent process or this protocol will require approval before being initiated.

6.4.2 Documentation of CM

• MPs (thick and thin blood smear; to facilitate rapid randomization and treatment initiation in Aim 2, a rapid diagnostic test at the bedside will be completed with a thick and thin blood smear obtained simultaneously)

•BCS ≤2
•Any other evident coma etiology *(e.g.* head trauma)

6.4.3 Medical History

• Active seizures-in the 24 hours before or after admission identified clinically or on routine EEG
• Creatinine.

6.4.4 Concomitant Treatments

• Note whether any AEDs were given in the 48 hours prior to enrollment and the doses of these if applicable
• Recent (within 14 days) or current enzyme-inducing agents including rifampin containing tuberculosis therapy or antiretroviral therapy for HIV infection (including protease inhibitors, nucleoside reverse transcriptase inhibitors, non-nucleoside reverse transcriptase inhibitors, integrase inhibitors or entry inhibitors)to be assessed as exclusion criteria

Given the plans for treatment cross over for patients failing their assigned treatment and the need to randomize to eLVT vs. phenobarbitone, additional exclusion criteria for years 2 and 3 when evaluating Aim 2 will include-

- Treatment within the past 12 hours with more than two doses of acute rescue treatment with a short-acting antiepileptic agent (Meaning > 0.4mg/kg IV diazepam; > 0.8 mg/kg PR diazepam; > 0.4 mg/kg IM paraldehyde)
- Treatment within the past 3 days with any long-acting anticonvulsant including phenobarbitone, phenytoin, or carbamazepine

6.4.5 Study Intervention Modifications

Upon recovery to a BCS ≥4, if the study participant is able to swallow based upon a bedside sips evaluation, the NGT will be removed and the study drug will be given orally. As per the methods detailed below, after the initial 8 study subjects' data are analyzed, a revised dosing schedule will be proposed, if necessary based upon the pharmacologic data. The cEEG will also be discontinued once a child is awake with a BCS ≥4.

6.4.6 Clinical Assessments

Physical Examination—This will include a bedside examination appropriate for any critically ill study participant. The CRF will include data added to the fitting chart as well as the BCS obtained. A physical examination is conducted twice daily at morning and evening ward rounds. Study participant will remain in the Pediatric Research Ward until discharge.

cEEG—Continuous EEG monitoring will be undertaken using microarray technology and MRI compatible electrodes. See Appendix E. cEEG interpretation will be provided on site by Drs. Birbeck, Mallewa and Postels for clinical management. For research purposes, these will be transmitted daily via a dedicated vsat using DropBox to Dr. Herman at BIDMC for a blinded analysis. She will only have the study participant's BCS and age.

Detailed Neurologic Assessment—this will include a detailed neurologic examination by a neurologist. Any notable abnormalities on the detailed neurologic assessment will be included in the CRF. This will be completed on admission, discharge and 7-days post eLVT administration.

6.4.7 Laboratory Evaluations

We will characterize laboratory adverse events according to a graded criteria (See Graded Toxicity Criteria in Protocol Appendix 1). These will be assessed for adverse events. All suspected LVT-associated adverse drug reactions should be recorded on CRFs. The protocol team should be notified of any Grade 3 LVT-associated AEs within 72 hours of the site being aware of the event along with site investigator plan for clinical management and follow up of these events as appropriate.

Grade 1 Toxicity: Continue LVT.

Grade 2 Toxicity: Continue LVT.

Grade 3 Toxicity: LVT should be held at the discretion of the Ward attending physician.

For abnormal laboratory results, repeat assessment/confirmation should be done as soon as possible. If repeat assessment confirms Grade 3 toxicity, continue to hold LVT and follow abnormal laboratory values weekly. All toxicities should continue to be monitored at least weekly until the toxicity is Grade 2 after which the subject will be removed from study.

Grade 4 Toxicity: LVT must be held if the SMC concludes this is possibly related to the study drug. For abnormal laboratory results, repeat assessment/confirmation should be done as soon as possible. All toxicities should continue to be monitored at least weekly until the toxicity is Grade 2 after which the subject will be removed from the study

6.4.8 Pharmacokinetic Studies

Pharmacokinetic assessments will be made upon children enrolled in dose escalation and the open label clinical trial. Predicted steady-state trough concentrations in a typical 2 year old with normal and moderate renal dysfunction (serum Cr=2.0) using a loading dose of 40mg/kg with 30mg/kg every 12 hours, fall within the desired range and are depicted in Strategy Figure 2(1)

Eight blood samples (0.7 mL) for LVT will be collected on each study subject: At time 0 (prior to first dose) 1.5, 4, 12 hr (prior to 2nd dose), 24hr (prior to 3rd dose), prior to and 4 hr post the 5th dose and 12 hrs after the last dose. Laboratory assessments will be made at baseline, 24 hours and 7 days post LVT administration. An ECG will be obtained at baseline

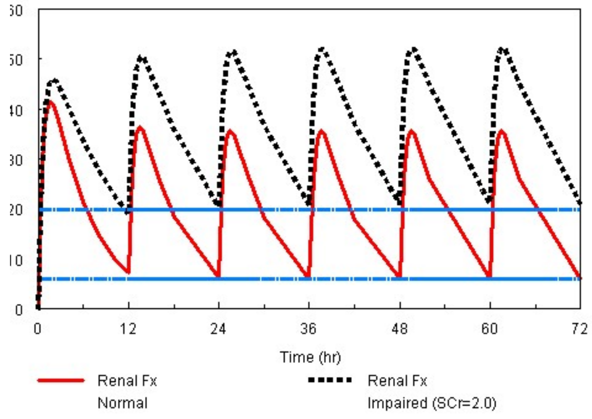

Figure 2: Predicted LVT Level for Initial Dose

determined by HPLC method (64). Pk data will be analyzed using both a mixed-effects population approach and empiric Bayesian approach, both performed with the computer program NONMEM. Plasma LVT levels will be summarized and evaluated after each dose-cohort.

7. MANAGEMENT OF ADVERSE EXPERIENCES

CM is associated with high rates of morbidity and mortality. The expected outcomes include severe anemia requiring blood transfusions, hyperlactatemia, acidosis, prolonged coma, hypoglycemia, severe thrombocytopenia, hyponatremia, extreme hyperpyrexia, seizures, and, neurologic sequelae including blindness, hemiparesis, hypotonia, and mutism. Death is also a common outcome occurring in 16% of pediatric CM patients on the QECH Research Ward over the past 5 years. All study subjects will receive treatment and management for these conditions consistent with the WHO's guidelines for the management of severe malaria(63)

Adverse Events related to the intervention may include aspiration, complications related to NGT placement and maintenance, vomiting, prolonged coma, and transient aggression or irritability upon regaining consciousness

Aspiration-Study subjects will be closely monitored clinically for signs of aspiration including Q4 hourly assessments of oxygen saturation. If oxygen saturations are below 95%, the ward attending will be notified to make a physician level assessment (auscultation, urgent PCV, etc.) and determine if aspiration is the potential underlying cause. If oxygen saturation declines and remains lower than the pre-aspiration baseline, the NGT will be placed to suction to remove any remaining gastric contents, the intervention will be discontinued and the NGT removed. Otherwise, the decision to discontinue the study drug intervention is at the discretion of the attending physician.

Complications of NGT placement and maintenance-If placement/maintenance of the NGT by experienced staff is difficult and result in nasal trauma and or significant epistaxis and the study subject is comatose and/or unable to swallow, the intervention will be discontinued and the NGT removed. Necessary measures to ameliorate any bleeding will be taken.

Vomiting-Although common in the early stages of malaria, once comatose, study participants with CM do not usually vomit. If vomiting occurs in comatose study participant, the NGT will be placed to suction to remove the remaining gastric contents, oxygen saturations will be assessed and once gastric contents have been removed, the NGT will be removed and the intervention will be discontinued.

Since prolonged coma and aggression/irritability due to LVT vs the underlying CM are impossible to distinguish on an individual patient basis, these potential AEs will be recorded, but will not serve as grounds for discontinuation of the study.

8. <u>CRITERIA FOR INTERVENTION DISCONTINUATION</u>

The intervention will be discontinued at the explicit request of the caregiver/guardian. The intervention will also be discontinued if based upon the BMP clinician's judgment LVT-associated adverse effects including vomiting, aspiration or complications related to administration of the study drug warrant discontinuation. A discontinuation form to be included in the CRF, will be completed by the attending physician, must be documented and filed for guardian-initiated withdrawals or discontinuation based upon AEs or elevated concerns regarding for potential AEs. Study participants who discontinue the intervention will remain on the Pediatric Research Ward for the high level care available on this unit.

We will characterize laboratory adverse events according to graded criteria per a modified version of the NINDS Common Data Elements for Adverse Events. Even though LVT has no clear safety signal based on laboratory abnormalities, this is a novel population for LVT use and laboratory assessment is required. All suspected adverse drug reactions (SADRs) will be recorded on CRFs. The core team will be notified of any Grade 3 SADR (including death) within 24 hours of the site being aware of the event along with the PI and clinician's plan for clinical management and follow up of these events as appropriate. Note that since children with CM are expected to have several abnormal laboratory parameters due to their underlying condition, a significant change from admission laboratory values will be the parameter assessed to identify laboratory-based SADRs. These are detailed in the Appendix.

Grade 1 Toxicity: Continue LVT.

Grade 2 Toxicity: Continue LVT.

Grade 3 Toxicity: LVT should be held at the discretion of the BMP clinical director. For abnormal laboratory results, repeat assessment/confirmation should be done as soon as possible. If repeat assessment confirms Grade 3 toxicity, continue to hold LVT and follow abnormal laboratory values weekly. All toxicities should continue to be monitored weekly until the toxicity is Grade 2.

Grade 4 Toxicity: LVT must be held if the adverse event is determined to be possibly related to LVT. For abnormal laboratory results, repeat assessment/confirmation should be done as soon as possible. If repeat assessment confirms Grade 4 toxicity, LVT should be permanently discontinued. If repeat assessment shows Grade 3 toxicity, continue to hold LVT and follow abnormal laboratory values. If toxicity resolves to ≤ Grade 2, LVT can be restarted. If > Grade 3 toxicity recurs after reintroduction of LVT, LVT must be permanently discontinued. All toxicities should continue to be monitored at least weekly until the toxicity is Grade 2 after which the subject will be removed from study.

9 STATISTICAL CONSIDERATIONS

9.1 Details of Randomization Process

The randomization process is designed to be blinded to data collectors. The steps for randomizing patients are:

1) A ordered list of random assignment to groups is generated.
    a. An open source SAS macro will be used to generate group assignment (Efird, 2011)
        i. The random number seeds will be pre-defined and stored.
        ii. Re-running the macro with the same random number seeds will replicate samelist of assignments.
        iii. SAS random number functions will generate the sequence of block size and the distribution of subjects within in each block.
        iv. Block sizes of 4 and 6 will be randomly selected.
        v. Random assignment of 100 subjects will be determined in advance.
2) The list of random assignments will be made available to clinical staff in two methods.
    a. The preferred method of accessing subject assignment will be through a link in the OpenClinica software.

i. At subject enrollment, staff will click on the link to determine which group the subject is assigned to.

b. As a backup method, sequentially numbered sealed opaque envelopes will be used for periods of time when the local OpenClinica is not available.

i. The envelopes will contain the same information as the link on OpenClinica and will only be made available as a back-up.

c. A paper log will track the process of subject enrollment.

i. As subjects enroll in the study, subject ID and sequential number will be logged.

ii. The sequential number of enrollment on the log will be used to determine which envelope will be used to assign randomization.

9.2 General Design Issues

We hypothesize that LVT use during acute CM will result in improved seizure control The initial study undertaken to address this hypothesis is a dose finding study of enteral LVT. LVT has been selected as the AED of choice due to its excellent side effect profile especially with regard to the lack of respiratory suppression even with loading doses of LVT. This is important since respiratory failure/apnea is a common mode of death from CM in hospitals in sub-Saharan Africa (where 90% of CM occurs) do not have recourse to mechanical ventilation. Enteral LVT has been chosen for its relative affordability and the feasibility of enteral LVT administration even in primary care settings in Africa. At this time, parental LVT treatment for a 15kg study participant for 3 days would be >$US300 and would be unaffordable in the public sector. In this dose escalation study, our primary goal is to determine the optimal dose for achieving seizure freedom in 75% of study participant with CM and seizures.

Our secondary aims are to

a. Assure feasibility and safety of eLVT
b. Obtain preliminary efficacy data for LVT as an anticonvulsant in acute pediatric CM through a open label, randomized assessment of optimal dose eLVT vs. standard AED treatment looking at time with seizure (in minutes) during the 72 hours after treatment allocation/consent.

9.3 Outcomes

9.3.1 Optimal dose of eLVT for seizure control in at least 75% of study participant with CM and seizures.

9.3.2 Secondary outcomes--Side effect/safety profile

a. Frequency of vomiting

b. Frequency of any aspiration

c. Frequency of aspiration requiring discontinuation of study drug

d. Frequency of NGT complications

e. Frequency of NGT complications requiring discontinuation and descriptions of events

f. Frequency of SADRs per laboratory assessments (hematologic, renal; and hepatic) at 1 and 7 days post eLVT administration compared to baseline parameters

g. Coma duration defined as time from admission to BCS>3

h. Aggression or irritability on regaining consciousness

i. Any other unanticipated adverse events which are not known to occur among study participant with CM and which may therefore be related to the interventions.

9.3.3 Open label, randomized clinical trial of optimal dose LVT vs. standard AED with the primary outcome of minutes with seizure per cEEG in the 72 hours after treatment allocation

Secondary evaluations will include

a. The AEDs required (including for breakthrough seizures in LVT group) during admission including agent(s) and overall quantity received
b. Mean time from admission to BCS≥4
c. Neurologic sequelae at discharge
d. Given that retinopathy status and pre-enrollment exposure to phenobarbitone may both impact LVT efficacy, two additional stratified analysis based upon these characteristics will be conducted as secondary analysis for Aim 2

9.4 Sample Size, Accrual and Analysis

<u>Dose Escalation Study:</u>
Sample: Up to 8 study participants administered one of four pre-specified doses of LVT, beginning with the standard dosing (100% standard) and subsequent doses at ~150%, 225% and 300% standard.
Endpoint: (1) seizure freedom in 75% of study participants for 24 hours after initiation of treatment, (2) toxicity, acute mortality.

Analysis: Our approach begins with the initial LVT loading dose d_1 in n_1 study participants. The target response is seizure freedom in 75% of study participants in 24 hours. Let Y_1 denote the number of study participants meeting the target response, and *p* the probability of response. If the estimate $\hat{p}$ of *p* is less than 0.75 we use the next higher dose d_2 in n_2 study participant and *p* is re-estimated. At any stage, if the estimate $\hat{p}$ ≥0.75 when we compute a 90% exact confidence interval (CI) for *p*(69), dose escalation is stopped. If the lower limit of the CI exceeds 0.50; otherwise, we use the next dose level. In this scenario an exact 90% CI for *p* based on 7 responses is (.529, .994) which meets the imposed condition (see Strategy Table 2). The probability that the half-width does not exceed 0.30 is 0.913 when $\hat{p}$ =0.75. (70) To refine subsequent estimation of *p* we will include Pk measurements x_1 in a dose-response logit model
$p = (1 + \exp(\beta_0 - \beta_1 d_1 - \beta_2 x_1))^{-1}$. (68)

Throughout the dose-escalation study we will also monitor for toxicity and acute mortality.

Endpoint or response: Toxicity, acute mortality
Although LVT is not expected to have a different safety profile than current AED treatments it behooves consideration of early stopping of the study should an unlikely event occur.
To frame our approach let *p* denote the event probability (eg, toxicity, mortality) in the LVT treatment group. Consider acute mortality (case fatality).The historic ward case fatality in this CM population is p_0 (=.16). If δ (>0) is the acceptable margin between LVT and standard AED then $H_0: p - p_0 \le \delta$ is regarded as acceptable, while $H_1: p - p_0 > \delta$ would be unacceptable. In words, if H_1 is true then LVT treatment shows case fatality a proportion δ above that of AED treatment, whereas under H_0 LVT is non-inferior to AED.

Table2: Exact confidence intervals for response probability *p*. *N*=8

# Responses	80% CI	90%CI
6	.462, .931	.400, .954
7	.594, .987	.529, .994
8	.750, 1.00	.688, 1.00

The dose escalation study is planned starting with 8 study participant at the standard dose (100% of standard) followed by 8 study participant at each of 150%, 225% and 300% of standard. We will use the accumulating data to judge the safety of LVT. Hence the total number of study participant at the end of all four dose levels in 32. Because of the small sample size we initially set δ=.08, for the first two sets of data (n=8, n=16) and then set δ=.04 for the second two sets of data (n=24 and n=32). If we use a significance level α=.10, exact *p*-values for testing H_0 vs H_1 and the 80% confidence limits (LCL, UCL) for *p* are shown I the table. The estimate of *p* is the proportion of fatalities among *n* study participant in treatment. The last line for each value of *n* gives the stopping boundary. For example suppose at the standard dose we see fewer than 4 deaths (50%); continuing to the next dose (if warranted), we will cease the study at this stage if the accumulated deaths exceeds 6 out of 16 treated (37.5%). We emphasize that these scenarios are not expected as LVT's safety profile is well established in different settings.

Table 3: Scenarios for stopping LVT treatment due to unexpected excess mortality

n	Limit, $p_0 + \delta$	Estimate,*p*	Exact p-value	Exact 80% LCL	Exact 80% UCL
8	0.24	0.3750	0.2967	0.1469	0.6554
8	0.24	0.5000	0.1004	0.2397	0.7603
16	0.24	0.3125	0.3341	0.1606	0.5035
16	0.24	0.3750	0.1641	0.2104	0.5654
16	0.24	0.4375	0.0657	0.2629	0.6250
24	0.20	0.2917	0.1889	0.1703	0.4416
24	0.20	0.3333	0.0892	0.2049	0.4845
32	0.20	0.2500	0.3018	0.1514	0.3742
32	0.20	0.3125	0.0898	0.2040	0.4400

<u>Summary of Escalation Plan</u>

We stop <u>for efficacy</u> when 7/8 study participants on a specific dose are seizure free for the 24 hours after LVT administration.

We stop <u>for toxicity</u> if mortality + grade 3 or 4 SADR exceeds the BMP baseline ward 16% allowing parameters as described above for small sample size and uncertainty

The stopping rules…
4 of 8 deaths/toxicity and we stop (50.0%)
6 of 16 deaths/toxicity and we stop (37.5%)
8 of 24 deaths/toxicity and we stop (33.3%)
9 of 32 deaths/toxicity and we stop (28.1%)

<u>Open Label, Randomized Comparison of optimal LVT vs. standard AED</u>
Sample: Two groups of 30 study participant (1) receiving the optimal LVT treatment identified in aim 1, or (2) receiving standard AED treatment.

Primary Endpoint: Minutes with seizure activity during the first 72 hours after initiation of treatment
Secondary Endpoints: (1) the use of additional chronic anticonvulsants for seizure control, (2) time to coma resolution defined as a Blantyre Coma Score (BCS) ≥ 4, (3) presence of neurologic sequelae at discharge, (4) acute mortality.

Analysis: The outcome data for the two-group design are $\{(Y_i, z_i, \mathbf{x}_i): 1 \le i \le n\}$ where Y_i is the response of the *i*-th study participant, z_i a treatment group indicator (z_i =1 for LVT, z_i =0 for standard) and $\mathbf{x}_i$ denotes additional explanatory variables (covariates) assessed prior to treatment initiation. Our analyses concern the mean response $E(Y_i \mid z_i, \mathbf{x}_i)$. Specifically, continuous responses (eg, minutes in seizure activity) will be analyzed on the original measurement scale or first transformed (logarithmic or square root) to mitigate skewness if appreciably present.(71) For a binary response (eg, mortality during treatment; presence of neurologic sequelae at discharge) we use a logit model $\log\left(\pi(z_i, \mathbf{x}_i) / (1 - \pi(z_i, \mathbf{x}_i))\right) = z_i\delta + \mathbf{x}_i'\boldsymbol{\beta}$ where $\pi(z_i, \mathbf{x}_i) = P[Y_i = 1 \mid z_i, \mathbf{x}_i]$ is the probability of response. The focus of inference is the coefficient δ for the indicator variable for LVT therapy; δ is the log-odds ratio for LVT versus standard treatment. The effect of LVT therapy on outcome can be assessed by testing $H_0: \delta = 0$.(72) In addition, we will provide an estimate and confidence interval for the estimated effect. Exact methods will be used wherever feasible. We will use survival analysis(73) (74)for time to coma resolution (BCS ≥4) because the coma resolution time might be right-censored in some study participant if the event was not observed. We will use a Cox regression model for the hazard $h(t \mid z_i, \mathbf{x}_i) = h_0(t)\exp(z_i\delta + \mathbf{x}_i'\boldsymbol{\beta})$ where δ is the log-hazard ratio for LVT versus standard treatment. The hypothesis of a null treatment effect is assessed by testing $H_0: \delta = 0$. Finally, in addition to the aforementioned analytic approaches we will also explore use of parametric models as well as Bayesian strategies. The latter places a prior distribution on $(\delta, \boldsymbol{\beta})$ and the basis for inference is the posterior distribution. Direct probability statements can be made on $(\delta, \boldsymbol{\beta})$. For the parameter of interest δ we will report the posterior mean, standard deviation and 95% credible and high probability density (HPD) intervals.(73, 75)

LVT is expected to have a positive effect on outcomes. Thus treatment effect is seen in an odds ratio ω< 1 for an undesirable event such as mortality or presence of neurologic sequelae at discharge, while ω>1 for a desirable event such as seizure freedom for 24 hours after treatment initiation. The power associated with a specified effect of LVT therapy on binary endpoints is shown in Strategy Table 3. The null hypothesis is $H_0: \omega = 1$.With standard AED treatment about 25% of study participant will be seizure

free for 24 hours after initiation, whereas with LVT treatment over 60% of study participant will have this outcome, that is ω >4.5. The power to detect this difference is over 79%. (70) If approximately 50% of study participant on standard AED treatment have neurologic sequelae at discharge, whereas with LVT approximately 17% of study participant are affected, that is $\omega \approx .20$, the power to detect this difference is near 79%. With a sample of 30 study participant in each group we realize that there is considerably less statistical power to detect smaller effect sizes. However, the purpose of aim 2 is to assess the magnitude of effect of LVT against standard AED treatment. For seizure freedom for 24 hours after initiation of treatment, to detect an effect ω >2.5 with 80% power will require 85 study participant per group assuming 25% of study participant under standard AED treatment have this endpoint.

Table 4: Power to detect an effect in test of $H_0: \omega = 1$ (two-sided α=.05)

Probability of outcome in standard, p_0	Odds Ratio, ω		
	**2.5**	**3.5**	**4.5**
0.25	.385	.636	.793
0.35	.415	.658	.802
0.45	.412	.640	.776
	**Odds Ratio, ω**		
	**0.2**	**0.3**	**0.4**
0.40	.723	.527	.361
0.50	.795	.589	.401
0.60	.837	.622	.417

Pk Study Statistical Power

Assuming that the inter-subject variability (% CV) for CL/F (clearance/bioavailability or fraction that is absorbed) will be approximately 50%[4], with 16-20, the mean pharmacokinetic parameters calculated in the study will have a 95% likelihood of being within 25% of the true population mean CL/F values. Subjects with incomplete pharmacokinetic data will be replaced. We estimate this will be the case with a small proportion of the study population. Complete pharmacokinetic data will be required on a likely maximum of 20 patients. With a sample size of 30, assuming a maximum sample size (full Pk data) on 26 and a mortality rate of 16% with early deaths resulting in incomplete data, we anticipate complete Pk data to be available on at least 20 study participants receiving the optimal dose. The results from subjects with incomplete data will still be included in Pk assessments.

[4] In healthy subjects the baseline %CV is substantially lower at 30% (See Glauser 2003)

9.5 Safety Monitoring

A local Study Monitoring Committee (SMC) will provide external oversight. The SMC chair will be contacted via telephone within 24 hours of any LVT-related adverse events (AE) resulting in discontinuation or death among study patients. The SMC will conduct a review of the study subject's record within 72 hours of the death using the paper records available on site. Weekly reports of LVT-related adverse events will be provided to the SMC in paper format before these data are available in the electronic database. A formal meeting of the SMC, which will include members of the research team for some portion of the meeting, will be held before each dose escalation in Aim 1 and at the end of each seasonal enrollment period. A final meeting during which a report will be generated will be held within 2 months of the enrollment completion. The SMC Chair may convene an *ad hoc* meeting at her discretion at any time. One member of the SMC with serve as the local liaison with the NINDS appointed Data Safety Monitoring Board (DSMB).

The Data Coordinating Center support unit will build a safety reporting dashboard into the OpenClinica platform. This work will begin in the mid-point of Year 1. It will be crafted to support the work of the safety monitor. Using the NINDS DSMB required reports, the dashboard will show at a glance the important study execution, data tracking and safety profile for the trial. The data managers will identify metadata and CDEs to populate reports and display on the dashboard. The dashboard will give PIs and safety monitoring committee members an up-to-date view of enrollment and safety/adverse events.

9.6 Data Analyses

Dose Finding Study

Potentially confounding variables include moderate to severe renal dysfunction, therefore this is an exclusion criteria. Also, the use of medication combinations that may cause complex and unpredictable drug-drug interactions could potentially confound the dose findings studies, therefore study participant on ATT and/or ARVs are also excluded. Study participant with incomplete PK data will be excluded from the dose-finding analysis, but their safety data will be included.

10. DATA COLLECTION, SITE MONITORING, AND ADVERSE EXPERIENCE REPORTING

10.1 Records to Be Kept

All laboratory specimens, evaluation forms, reports, images obtained, and other records that leave the site will be identified only by the study ID number to maintain subject confidentiality. All paper records will be kept in a locked file cabinet. All computer entry and networking programs will be done using IDs only. Clinical information will not be released without written permission of the subject, except as necessary for monitoring by IRB, the NINDS, the OHRP, the Malawi Pharmacy, Medicines and Poisons Board (PMPB), the sponsor, or the sponsor's designee. To enable evaluations and/or audits from Regulatory Authorities and NINDS or its designees, the investigators will keep records, including the identification of all medical charts and associated source documents and copies of all CRFs. The investigators will contact NINDS before disposing of any such materials.

10.2 Role of Data Management

10.2.1 The clinical site will assure that all paper records capturing the required are entered into the OpenClinica online system daily, as allowed by internet availability. Daily electronic data back-ups will be maintained locally regardless of internet accessibility. Paper records will be maintained on site in a locked storage facility within the Malaria Alert Center.

10.2.2 BRIC, the Data Coordinating Center for this project, will develop the appropriate forms for data collection on paper to harmonize with data entry using, as much as is feasible, NINDS CDEs. BRIC will support a data collection protocol in which data will be entered in Malawi, uploaded to a central spot. Native to OPENCLINICA are some powerful data management routines to find and report discrepancies (dirty data) to data managers for editing. It is this platform that is the heart of our DCC operations. The Malawi data managers/entry personnel will enroll subjects, track their visits and enter case report form data into OpenClinica. Periodically, when internet service is available, data will be exported from OpenClinica and sent to East Lansing. The East Lansing data manager will receive the exported file and merge/interleave the records into OpenClinica. All reporting and analysis will be from data resident in OpenClinica. We will develop an automated script to export data to East Lansing. The MSU/BRIC programmer supervisor will oversee the development of the data integration script and data reporting/discrepancy management programs. The MSU/BRIC data manager will run the data integration script, review uploaded data and communicate discrepancies with the Malawi based data manager.

Modern data entry/management platforms require keen attention to the details of metadata. The BRIC data manager will carefully hone and craft the metadata to deliver data entry forms and annotate dataset for analysis and assure conformance to NINDS CDEs when possible. In defining variables we will put into place the beginnings of a common library of forms that honor NINDS CDEs and procedures that follow GCDMP principles. For ease of entry and clinician simplicity, adverse event descriptions are typically written in plain text. To categorize these entries the text requires coding. NINDS CDEs indicate that MEDDra is a possible dictionary to which these texts can be coded or standardized. BRIC runs a significant coding operation. We propose to add the MEDDra dictionary to the BRIC coding library; and for this protocol begin coding adverse event texts to it. In the process of coding open/free text descriptions there will be entries that require clarification or new additions to the MEDDra dictionary, with the GCDMP guidelines source dictionary management is defined as a key role of data management operations. BRIC has a history of coding verbatim terms to standard dictionaries. We use Current Procedural Terminology (CPT) for medical procedures and International Classification of Disease (ICD) for comorbidities. For this protocol we will plan a coding and dictionary management platform for any sister trials or fuller Phase II trials that can be run independently in Malawi.

10.3 Quality Assurance

Prior to the initiation of the study, training sessions will be held Malaria Alert Center with clinician co-investigators, Research Ward staff, the investigators and their study coordinators for the study. This meeting will include a detailed discussion of the protocol, performance of study procedures, CRF completion, simulation of study procedures and specimen collection methods, as applicable.

After completion of the entry process, computer logic checks will be run to check for such items as inconsistent study dates and outlying laboratory values. Any necessary correction will be made to the database and documented via addenda or audit trail. A manual review of selected line listings will also be performed at the end of the study.

The Project Coordinator will assist the PI in assuring protocol compliance, ethical standards, regulatory compliance and data quality at the clinical site. The coordinator will provide real time daily review of all

study files to assure protocol compliance and documentation completeness. The study sites may also be subject to quality assurance audits by the NINDS or its designees and appropriate regulatory agencies. Audits may include reviews of paper or electronic records.

10.4 Adverse Experience Reporting

The AEs of Special Interest not otherwise explained by the patient's underlying illness will be submitted in writing to the SMC Chair or her SMC member designee within one week of occurrence. Serious AEs, defined as those resulting in discontinuation of the study drug, will be reported within 24 hours of occurrence. Local IRB guidelines will also be followed.

AEs to be submitted to the SMC within 24 hours of occurrence:

1. Death
2. Vomiting, aspiration or NGT complications resulting in discontinuation of the intervention

11. HUMAN SUBJECTS

11.1 Institutional Review Board (IRB) Review and Informed Consent

This protocol, the informed consent document, and any subsequent modifications must be reviewed and approved by the IRB or Ethics Committee (EC) responsible for oversight of the study. Written informed consent must be obtained from the parents or legal guardians of subjects. The informed consent will describe the purpose of the study, the procedures to be followed, and the risks and benefits of participation. A copy of the consent form will be given to the subject (or parent or legal guardian).

11.2 Subject Confidentiality

All laboratory specimens, evaluation forms, reports, EEGs, and other records that leave the site will be identified only by the study ID number to maintain subject confidentiality. All records will be kept in a locked file cabinet. All computer entry and networking programs will be done using SIDs only. Clinical information will not be released without written permission of the subject, except as necessary for monitoring by BIRB, COMREC, the FDA, the Malawi Medicines and Poisons Board, the NINDS, the OHRP, the sponsor, or the sponsor's designee.

11.3 Study Modification/Discontinuation

The study may be modified or discontinued at any time by the IRB, the NINDS, the sponsor, the OHRP, the FDA, the Malawi Medicines and Poisons Board or other government agencies as part of their duties to ensure that research subjects are protected.

12. PUBLICATION OF RESEARCH FINDINGS

ALL MANUSCRIPTS, ABSTRACTS, AND POSTER SUBMISSIONS MUST BE SUBMITTED TO THE EXECUTIVE COMMITTEE[5] FOR APPROVAL. THE PRIMARY OUTCOME OF THIS STUDY WILL BE SUBMITTED

[5] Publications executive committee includes: GL Birbeck, TE Taylor, E Capparelli

FOR PUBLICATION NO LATER THAN 1 YEAR AFTER STUDY COMPLETION. FINANCIAL SUPPORT FROM THE NINDS WILL BE ACKNOWLEDGED IN ALL PUBLICATIONS.

Addendum to Protocol "A DOSE-ESCALATION, SAFETY AND FEASIBILITY STUDY OF ENTERAL LEVETIRACETAM FOR SEIZURE CONTROL IN PEDIATRIC CEREBRAL MALARIA" Version 10.2

ALL DATA THAT LEAVES THE MALAWI STUDY SITE IS DE-IDENTIFIED (SO RECORDS UPLOADED INTO THE DATABASE MANAGED BY MSU, DATA SENT TO SUE AT BIDMC AND DATA SENT TO EDMUND AT UCSD). THERE IS NO "KEY" TO STUDY ID…JUST HARD COPY PATIENT RECORDS ON SITE IN MALAWI. FACULTY FROM MSU AND BIDMC ACTUALLY COME TO MALAWI PERIODICALLY TO ASSIST WITH SET UP, SYSTEMS ASSESSMENTS, ETC. AND IN THAT ROLE, THEY MAY VERY WELL INTERACT WITH THE PATIENTS AND/OR COME ACROSS THE IDENTIFIABLE DATA

REFERENCES

1. Pellock JM, Glauser TA, Bebin EM, Fountain NB, Ritter FJ, Coupez RM, et al. Pharmacokinetic study of levetiracetam in study participant. Epilepsia. 2001 Dec;42(12):1574-9.
2. Teja-Isavadharm P, Watt G, Eamsila C, Jongsakul K, Li Q, Keeratithakul G, et al. Comparative pharmacokinetics and effect kinetics of orally administered artesunate in healthy volunteers and patients with uncomplicated falciparum malaria. Am J Trop Med Hyg. 2001 Dec;65(6):717-21.
3. Haldar K, Murphy SC, Milner DA, Taylor TE. Malaria: mechanisms of erythrocytic infection and pathological correlates of severe disease. Annu Rev Pathol. 2007;2:217-49.
4. Lukyanetz EA, Shkryl VM, Kostyuk PG. Selective blockade of N-type calcium channels by levetiracetam. Epilepsia. 2002 Jan;43(1):9-18.
5. Lynch BA, Lambeng N, Nocka K, Kensel-Hammes P, Bajjalieh SM, Matagne A, et al. The synaptic vesicle protein SV2A is the binding site for the antiepileptic drug levetiracetam. Proc Natl Acad Sci U S A. 2004 Jun 29;101(26):9861-6.
6. Jukes M, Pinder M, Grigorenko E, Smith H, Walraven G, Bariau E, et al. Long-tern impact of malaria chemoprophylaxis on cognitive abilities and educational attainment: Follow-up of a controlled trial. PLOS Clinical Trials. 2006;1(4):e19.
7. Loscher W, Honack D, Rundfeldt C. Antiepileptogenic effects of the novel anticonvulsant levetiracetam (ucb L059) in the kindling model of temporal lobe epilepsy. J Pharmacol Exp Ther. 1998;284(2):474-9.
8. Ji-qun C, Ishihara K, Nagayama T, Serikawa T, Sasa M. Long-lasting antiepileptic effects of levetiracetam against epileptic seizures in the spontaneously epileptic rat (SER): differentiation of levetiracetam from conventional antiepileptic drugs. Epilepsia. 2005 Sep;46(9):1362-70.
9. Ohno Y, Ishihara S, Terada R, Kikuta M, Sofue N, Kawai Y, et al. Preferential increase in the hippocampal synaptic vesicle protein 2A (SV2A) by pentylenetetrazole kindling. Biochem Biophys Res Commun. 2009 Dec 18;390(3):415-20.
10. DRUGDEX. Levetiracetam.: Greenwood Village.
11. Crawley J, Waruiru C, Mithwani S, Mwangi I, Watkins W, Ouma D, et al. Effect of phenobarbital on seizure frequency and mortality in study participanthood cerebral malaria: a randomised, controlled intervention study. Lancet. 2000;355(9205):701-6.
12. Shinnar S, Glauser TA. Febrile seizures. J Study participant Neurol. 2002;17 Suppl 1:S44-52.
13. Glauser TA, Mitchell WG, Weinstock A, Bebin M, Chen D, Coupez R, et al. Pharmacokinetics of levetiracetam in infants and young study participant with epilepsy. Epilepsia. 2007 Jun;48(6):1117-22.
14. Koukkari MW, Guarino EJ. Retrospective study of the use of levetiracetam in study participanthood seizure disorders. J Study participant Neurol. 2004 Dec;19(12):944-7.
15. Kirmani BF, Crisp ED, Kayani S, Rajab H. Role of intravenous levetiracetam in acute seizure management of study participant. Pediatr Neurol. 2009 Jul;41(1):37-9.
16. Gallentine WB, Hunnicutt AS, Husain AM. Levetiracetam in study participant with refractory status epilepticus. Epilepsy Behav. 2009 Jan;14(1):215-8.
17. Goraya JS, Khurana DS, Valencia I, Melvin JJ, Cruz M, Legido A, et al. Intravenous levetiracetam in study participant with epilepsy. Pediatr Neurol. 2008 Mar;38(3):177-80.
18. Reiter P, Huff A, Knupp K, Valuck R. Intravenous Levetiracetam in the Management of Acute Seizures in Study participant. Pediatric Neurology. 2010;43(2):117-21.
19. Depositario-Cabacar DT, Peters JM, Pong AW, Roth J, Rotenberg A, Riviello JJ, Jr., et al. High-dose intravenous levetiracetam for acute seizure exacerbation in study participant with intractable epilepsy. Epilepsia. 2010 Feb 12;51(7):1319-22.
20. Alehan F, Ozcay F, Haberal M. The use of levetiracetam in a study participant with nonconvulsive status epilepticus. J Study participant Neurol. 2008 Mar;23(3):331-3.
21. Abend NS, Gutierrez-Colina AM, Topjian AA, Zhao H, Guo R, Donnelly M, et al. Nonconvulsive seizures are common in critically ill study participant. Neurology. Mar 22;76(12):1071-7.

22. Ramantani G, Ikonomidou C, Walter B, Rating D, Dinger J. Levetiracetam: safety and efficacy in neonatal seizures. Eur J Paediatr Neurol. Jan;15(1):1-7.
23. Ng YT, Hastriter EV, Cardenas JF, Khoury EM, Chapman KE. Intravenous levetiracetam in study participant with seizures: a prospective safety study. J Study participant Neurol. May;25(5):551-5.
24. Patsalos PN. Clinical pharmacokinetics of levetiracetam. Clin Pharmacokinet. 2004;43(11):707-24.
25. Bialer M, Johannessen SI, Kupferberg HJ, Levy RH, Loiseau P, Perucca E. Progress report on new antiepileptic drugs: a summary of the fifth eilat conference (EILAT V) [In Process Citation]. Epilepsy Res. 2001;43(1):11-58.
26. Obeid M, Ponga A. Efficacy and tolerability of high oral doses of Levetiracetam in study participant with epilepsy. Epilepsy Research. 2010;91:101-5.
27. French J, Edrich P, Cramer JA. A systematic review of the safety profile of levetiracetam: a new antiepileptic drug. Epilepsy Res. 2001 Nov;47(1-2):77-90.
28. Briggs DE, French JA. Levetiracetam safety profiles and tolerability in epilepsy patients. Expert Opin Drug Saf. 2004 Sep;3(5):415-24.
29. Tripathi M, Vibha D, Choudhary N, Prasad K, Srivastava MV, Bhatia R, et al. Management of refractory status epilepticus at a tertiary care centre in a developing country. Seizure. Mar;19(2):109-11.
30. Grants.Trials.gov. Safety and Tolerability Study of Levetiracetam to Treat Patients With Status Epilepticus.
31. WHO. Mental Health Gap Action Programme. 2008. p. http://www.who.int/entity/mental_health/mhgap_final_english.pdf.
32. Birbeck G, Molyneux M, Seydel PKK, Chimalizeni Y, Kawaza K, Taylor T. The Blantyre Malaria Project Epilepsy Study (BMPES): Neurologic Outcomes in a Prospective Exposure-Control Study of Retinopathy-Positive Pediatric Cerebral Malaria Survivors. Lancet Neurology. 2010;9(12):1173-81.
33. Newton CR, Warrell DA. Neurological manifestations of falciparum malaria. Ann Neurol. 1998;43(6):695-702.
34. Newton CR, Hien TT, White N. Cerebral malaria. J Neurol Neurosurg Psychiatry. 2000;69(4):433-41.
35. Newton P, White N. Malaria: new developments in treatment and prevention. Annu Rev Med. 1999;50:179-92.
36. Dondorp AM, Fanello CI, Hendriksen IC, Gomes E, Seni A, Chhaganlal KD, et al. Artesunate versus quinine in the treatment of severe falciparum malaria in African study participant (AQUAMAT): an open-label, randomised trial. Lancet. Nov 13;376(9753):1647-57.
37. Molyneux ME, Taylor TE, Wirima JJ, Borgstein A. Clinical features and prognostic indicators in paediatric cerebral malaria: a study of 131 comatose Malawian study participant [see comments]. Q J Med. 1989;71(265):441-59.
38. Slutsker L, Taylor TE, Wirima JJ, Steketee RW. In-hospital morbidity and mortality due to malaria-associated severe anaemia in two areas of Malawi with different patterns of malaria infection. Trans R Soc Trop Med Hyg. 1994 Sep-Oct;88(5):548-51.
39. Newton CR, Taylor TE, Whitten RO. Pathophysiology of fatal falciparum malaria in African study participant. Am J Trop Med Hyg. 1998;58(5):673-83.
40. Newton CR, Chokwe T, Schellenberg JA, Winstanley PA, Forster D, Peshu N, et al. Coma scales for study participant with severe falciparum malaria. Trans R Soc Trop Med Hyg. 1997;91(2):161-5.
41. Marsh K, Forster D, Waruiru C, Mwangi I, Winstanley M, Marsh V, et al. Indicators of life-threatening malaria in African study participant. N Engl J Med. 1995 May 25;332(21):1399-404.
42. Crawley J, Smith S, Kirkham F, Muthinji P, Waruiru C, Marsh K. Seizures and status epilepticus in study participanthood cerebral malaria. Qjm. 1996;89(8):591-7.
43. Carme B, Bouquety JC, Plassart H. Mortality and sequelae due to cerebral malaria in African study participant in Brazzaville, Congo. Am J Trop Med Hyg. 1993;48(2):216-21.
44. Molyneux ME, Taylor TE, Wirima JJ, Borgstein A. Clinical features and prognostic indicators in paediatric cerebral malaria: a study of 131 comatose Malawian study participant. Q J Med. 1989;71(265):441-59.

45. Bondi FS. The incidence and outcome of neurological abnormalities in study participanthood cerebral malaria: a long-term follow-up of 62 survivors. Trans R Soc Trop Med Hyg. 1992;86(1):17-9.
46. Meremikwu MM, Asindi AA, Ezedinachi E. The pattern of neurological sequelae of study participanthood cerebral malaria among survivors in Calabar, Nigeria. Cent Afr J Med. 1997;43(8):231-4.
47. van Hensbroek MB, Palmer A, Jaffar S, Schneider G, Kwiatkowski D. Residual neurologic sequelae after study participanthood cerebral malaria. J Pediatr. 1997;131(1 Pt 1):125-9.
48. Brewster DR, Kwiatkowski D, White NJ. Neurological sequelae of cerebral malaria in study participant. Lancet. 1990;336(8722):1039-43.
49. Bangirana P, Nakasujja N, Giordani B, Opoka RO, John CC, Boivin MJ. Reliability of the Luganda version of the Study participant Behaviour Checklist in measuring behavioural problems after cerebral malaria. Study participant Adolesc Psychiatry Ment Health. 2009;3:38.
50. Boivin MJ, Bangirana P, Byarugaba J, Opoka RO, Idro R, Jurek AM, et al. Cognitive impairment after cerebral malaria in study participant: a prospective study. Pediatrics. 2007 Feb;119(2):e360-6.
51. Taylor TE, Fu WJ, Carr RA, Whitten RO, Mueller JS, Fosiko NG, et al. Differentiating the pathologies of cerebral malaria by postmortem parasite counts. Nat Med. 2004 Feb;10(2):143-5.
52. Beare NA, Harding SP, Taylor TE, Lewallen S, Molyneux ME. Perfusion abnormalities in study participant with cerebral malaria and malarial retinopathy. J Infect Dis. 2009 Jan 15;199(2):263-71.
53. Beare NA, Taylor TE, Harding SP, Lewallen S, Molyneux ME. Malarial retinopathy: a newly established diagnostic sign in severe malaria. Am J Trop Med Hyg. 2006 Nov;75(5):790-7.
54. Lewallen S, Bronzan RN, Beare NA, Harding SP, Molyneux ME, Taylor TE. Using malarial retinopathy to improve the classification of study participant with cerebral malaria. Trans R Soc Trop Med Hyg. 2008 Nov;102(11):1089-94.
55. Lewallen S, Harding SP, Ajewole J, Schulenburg WE, Molyneux ME, Marsh K, et al. A review of the spectrum of clinical ocular fundus findings in P. falciparum malaria in African study participant with a proposed classification and grading system. Trans R Soc Trop Med Hyg. 1999;93(6):619-22.
56. Lewallen S, White VA, Whitten RO, Gardiner J, Hoar B, Lindley J, et al. Clinical-histopathological correlation of the abnormal retinal vessels in cerebral malaria. Arch Ophthalmol. 2000;118(7):924-8.
57. Birbeck G, Team BS. Neurologic morbidity among cerebral malaria survivors—A call for neuroprotective clinical trials. 133rd Annual Meeting for the American Neurological Association; 2008 September 21-24.; Salt Lake City, UT; 2008. p. 7.
58. Birbeck G, Molyneux M, Kaplan P, Mwandira E, Sapuwa M, Jafuli M, et al. Neurologic outcomes among cerebral malaria survivors: The Blantyre Malaria Project Epilepsy Study. . Neurology. 2008;70(Suppl1):A100-A1.
59. Crawley J, Smith S, Muthinji P, Marsh K, Kirkham F. Electroencephalographic and clinical features of cerebral malaria. Arch Dis Study participant. 2001;84(3):247-53.
60. Birbeck G, Taylor T, Molyneux M, Mwandira E, Jalfuli M, Kaplan P. EEG Correlates in Acute Cerebral Malaria American Clinical Neurophysiology Society Annual Meeting; 2008; Savannah; 2008. p. Abstract book page 23.
61. Carter JA, Neville BG, White S, Ross AJ, Otieno G, Mturi N, et al. Increased prevalence of epilepsy associated with severe falciparum malaria in study participant. Epilepsia. 2004 Aug;45(8):978-81.
62. Potchen MJ, Birbeck GL, Demarco JK, Kampondeni SD, Beare N, Molyneux ME, et al. Neuroimaging findings in study participant with retinopathy-confirmed cerebral malaria. Eur J Radiol. 2009 Apr 2.
63. WHO. Guidelines for the Treatment of Malaria. Second ed. Genev: WHO Press; 2010.
64. Ratnaraj N, Doheny HC, Patsalos PN. A micromethod for the determination of the new antiepileptic drug levetiracetam (ucb LO59) in serum or plasma by high performance liquid chromatography. Ther Drug Monit. 1996 Apr;18(2):154-7.
65. O'Quigley J, Chevret S. Methods for dose finding studies in cancer clinical trials: a review and results of a Monte Carlo study. Stat Med. 1991 Nov;10(11):1647-64.

66. O'Quigley J, Pepe M, Fisher L. Continual reassessment method: a practical design for phase 1 clinical trials in cancer. Biometrics. 1990 Mar;46(1):33-48.
67. O'Quigley J, Shen LZ. Continual reassessment method: a likelihood approach. Biometrics. 1996 Jun;52(2):673-84.
68. Piantadosi S, Liu G. Improved designs for dose escalation studies using pharmacokinetic measurements. Stat Med. 1996 Aug 15;15(15):1605-18.
69. Fleiss J, Levin B, Paik M. Statistical Methods for Rates and Proportions. 3rd ed. New York, NY: Wiley-Interscience; 2003.
70. SAS/STAT User's Guide, The Power Procedure. 9.2 ed. Cary, NC: SAS Institute Inc; 2010.
71. Gardiner JC, Luo Z, Roman LA. Fixed effects, random effects and GEE: what are the differences? Stat Med. 2009 Jan 30;28(2):221-39.
72. Gardiner J, Luo Z. Logit Models in |Practice: B, C, E, G, M, N, O... SAS Global Forum; 2011; Las Vegas, NV; 2011. p. 341.
73. Gardiner J. Survival Analysis: Overview of Parametric, Nonparametric, and Semiparametric approaches and New Developments. SAS Global Forum; 2010; Seattle, WA; 2010. p. 252.
74. Marubini E, Valsecchi M. Analysing Survival Data from Clinical Trials and Observational Studies. Chichester, England: John Wiley & Sons, Inc.; 1995.
75. Ibrahim J, chen M, Sinha D. Bayesian Survival Analysis. New York: Springer-Verlag; 2001.
76. Efird J. Blocked Randomization with Randomly Selected Block Sizes *Int. J. Environ. Res. Public Health* 2011, *8*, 15-20; doi:10.3390/ijerph8010015

Protocol Appendix1:
GRADED TOXICITY CRITERIA

ABBREVIATIONS: Abbreviations utilized in the Table:

ULN = Upper Limit of Normal	LLN = Lower Limit of Normal
R_x = Therapy	Req = Required
Mod = Moderate	IV = Intravenous
ADL = Activities of Daily Living	Dec = Decreased

ESTIMATING SEVERITY GRADE

For abnormalities NOT found elsewhere in the Toxicity Tables use the scale below to estimate grade of severity:

GRADE 1 **Mild** Transient or mild discomfort (< 48 hours); no medical intervention/therapy required

GRADE 2 **Moderate** Mild to moderate limitation in activity - some assistance may be needed; no or minimal medical intervention/therapy required

GRADE 3 **Severe** Marked limitation in activity, some assistance usually required; medical intervention/therapy required, hospitalizations possible

GRADE 4 **Life-threatening** Extreme limitation in activity, significant assistance required; significant medical intervention/therapy required, hospitalization or hospice care probable

GRADE 5 **Death**

SERIOUS OR LIFE-THREATENING AEs

ANY clinical event deemed by the clinician to be serious or life-threatening should be considered a grade 4 event. Clinical events considered to be serious or life-threatening include, but are not limited to: tetany, diabetic ketoacidosis, disseminated intravascular coagulation, diffuse petechiae, and paralysis

COMMENTS REGARDING THE USE OF THESE TABLES

- Standardized and commonly used toxicity tables (Division of AIDS, NCI's Common Toxicity Criteria (CTC), and World Health Organization (WHO)) have been adapted for use by the Division of Microbiology and Infectious Diseases (DMID) and modified to better meet the needs of participants in DMID trials.
- For parameters not included in the following Toxicity Tables, sites should refer to the "Guide For Estimating Severity Grade" located above.
- Criteria are generally grouped by body system.
- Some protocols may have additional protocol specific grading criteria, which will supersede the use of these tables for specified criteria

**LOCAL REACTIONS**				
Induration	< 10mm	10-25 mm	26-50mm	>50mm
Erythema	< 10mm	10-25 mm	26-50mm	>50mm
Edema	< 10mm	10-25 mm	26-50mm	>50mm
Rash at Injection Site	< 10mm	10-25 mm	26-50mm	>50mm
Pruritus	Slight itching at injection site	Moderate itching at injection extremity	Itching at injection extremity and other sites	Itching over entire body
**GASTROINTESTINAL**				
	**GRADE 1**	**GRADE 2**	**GRADE 3**	**GRADE 4**
AST (SGOT)	1.1 - <2.0 x ULN	2.0 – <3.0 x ULN	3.0 – 8.0 x ULN	> 8 x ULN
ALT (SGPT)	1.1 - <2.0 x ULN	2.0 – <3.0 x ULN	3.0 – 8.0 x ULN	> 8 x ULN
GGT	1.1 - <2.0 x ULN	2.0 – <3.0 x ULN	3.0 – 8.0 x ULN	> 8 x ULN
**HEMATOLOGY**				
	**GRADE 1**	**GRADE 2**	**GRADE 3**	**GRADE 4**
Absolute Neutrophil Count	750-1200/mm^3	400-749/mm^3	250-399/mm^3	<250/mm^3

ELECTROLYTES				
	**GRADE 1**	**GRADE 2**	**GRADE 3**	**GRADE 4**
Hypernatremia		145-149 mEq/L	150-155 mEq/L	>155 mEq/L or abnormal sodium
Hyperkalemia	5.0-5.9 mEq/L	6.0-6.4 mEq/L	6.5-7.0 mEq/L	>7.0 mEq/L or abnormal potassium AND cardiac arrhythmia
Hypokalemia	3.0-3-5 mEq/L	2.5-2.9 mEq/L	2.0-2.4 mEq/L	<2.0 mEq/L or abnormal potassium AND cardiac arrhythmia
Hyperglycemia	116-159 mg/dL	160-249 mg/dL	250-400 mg/dL	>400 mg/dL or ketoacidosis

FOR LABORATORY PARAMETERS IMPACTED BY CEREBRAL MALARIA WHICH ARE EXPECTED TO BE DISORDERED AT BASELINE (PRIOR TO LVT ADMINISTRATION)

For evaluation at 24 hours post LVT Administration

	GRADE 1	GRADE 2	GRADE 3	GRADE 4
Bilirubin	No improvement from baseline	20% increase from baseline	30% increase from baseline	50% increase from baseline
Hemoglobin	Not applicable as ongoing anemia and need for blood transfusions not unexpected.			
Platelets	No improvement from baseline	20% decrease from baseline	30% decrease from baseline	50% decrease from baseline
Serum sodium	No improvement from baseline	20% decrease from baseline	30% decrease from baseline	50% decrease from baseline
Serum glucose	Not applicable as hypoglycemia during first 72 hours after admission with CM is not unexpected and is closely monitored for.			

For evaluation at 7 days post LVT Administration

	GRADE 1	GRADE 2	GRADE 3	GRADE 4
Bilirubin	No improvement from 24 hours post LVT	20% increase from 24 hours post LVT	30% increase from 24 hours post LVT	50% increase from 24 hours post LVT
Hemoglobin	No improvement from baseline but increased reticulocytes	No improvement from baseline and no increase in reticulocytes	Decreased from last measured but increased reticulocytes	Decreased from last measured and no increase in reticulocytes
Platelets	-------------------	50,000-75,000/mm^3	25,000-49,999/mm^3	<25,000/mm^3
Hypernatremia		145-149 mEq/L	150-155 mEq/L	>155 mEq/L or abnormal sodium
Hyponatremia		130-135 mEq/L	129-124 mEq/L	<124 mEq/L or abnormal sodium

**OTHER**				
	**GRADE 1**	**GRADE 2**	**GRADE 3**	**GRADE 4**
Allergy	Pruritus without Rash	Pruritic Rash	Mild Urticaria	Severe Urticaria Anaphylaxis, Angi-oedema
Cutaneous	Localized rash	Diffuse macu-lopapular Rash	Generalized urti-caria	Stevens-Johnson Syndrome or Ery-thema multiforme
Stomatitis	Mild discomfort	Painful, difficulty swallowing, but able to eat and drink	Painful: unable to swallow solids	Painful: unable to swallow liquids; re-quires IV fluids
Clinical symptoms *not otherwise specified* in this table	No therapy; moni-tor condition	May require mini-mal intervention and monitoring	Requires medical care and possible hospitalization	Requires active medical intervention, hospitalization, or hospice care
Laboratory values *not otherwise specified* in this table	Abnormal, but re-quiring no immedi-ate intervention; follow	Sufficiently abnor-mal to require evaluation as to causality and per-haps mild thera-peutic interven-tion, but not of suf-ficient severity to warrant immediate changes in study drug	Sufficiently severe to require evalua-tion and treat-ment, including at least temporary suspension of study drug	Life-threatening se-verity; Requires im-mediate evaluation, treatment, and usu-ally hospitalization; Study drug must be stopped immediately and should not be restarted until the abnormality is clearly felt to be caused by some other mechanism that study drug

Protocol Appendix 2:

Standard AED Treatment for acute provoked seizures

Diazepam 0.2mg/kg iv or 0.4mg/kg pr

Repeat in 10 minutes if no response

If paraldehyde is available, acute management my incorporate this agent rather than diazepam.

Paraldehyde: 0.2 ml/kg, IM.

Repeat in 10 minutes if no response.

After diazepam or paraldehyde, if no response or the acute seizure(s) that required treatment was prolonged and or recurrent give phenobarbitone 20mg/kg IV followed by 10mg/kg/day divided BDS

If phenobarbitone fails to control ongoing seizures or respiratory issues are a concern, phenytoin is to be given/added at 18mg/kg IV followed by 10mg/kg/divided BDS.
