# Supplementary material for: A clinical trial of enteral Levetiracetam for acute seizures in pediatric cerebral malaria
Source: BMC Pediatr. 2019 Nov 1;19:399. doi: 10.1186/s12887-019-1766-2 (PMC6824014; doi:10.1186/s12887-019-1766-2)
Supplement: Supplementary file 2 — Additional file 2. Graded toxicity Criteria [file 12887_2019_1766_MOESM2_ESM.pdf]

GRADED TOXICITY CRITERIA

ABBREVIATIONS: Abbreviations utilized in the Table:

ULN = Upper Limit of Normal	LLN = Lower Limit of Normal
R_x = Therapy	Req = Required
Mod = Moderate	IV = Intravenous
ADL = Activities of Daily Living	Dec = Decreased

ESTIMATING SEVERITY GRADE

For abnormalities NOT found elsewhere in the Toxicity Tables use the scale below to estimate grade of severity:

GRADE 1 **Mild** Transient or mild discomfort (< 48 hours); no medical intervention/therapy required

GRADE 2 **Moderate** Mild to moderate limitation in activity - some assistance may be needed; no or minimal medical intervention/therapy required

GRADE 3 **Severe** Marked limitation in activity, some assistance usually required; medical intervention/therapy required, hospitalizations possible

GRADE 4 **Life-threatening** Extreme limitation in activity, significant assistance required; significant medical intervention/therapy required, hospitalization or hospice care probable

GRADE 5 **Death**

SERIOUS OR LIFE-THREATENING AEs

ANY clinical event deemed by the clinician to be serious or life-threatening should be considered a grade 4 event. Clinical events considered to be serious or life-threatening include, but are not limited to: tetany, diabetic ketoacidosis, disseminated intravascular coagulation, diffuse petechiae, and paralysis

COMMENTS REGARDING THE USE OF THESE TABLES

- Standardized and commonly used toxicity tables (Division of AIDS, NCI's Common Toxicity Criteria (CTC), and World Health Organization (WHO)) have been adapted for use by the Division of Microbiology and Infectious Diseases (DMID) and modified to better meet the needs of participants in DMID trials.
- For parameters not included in the following Toxicity Tables, sites should refer to the "Guide For Estimating Severity Grade" located above.
- Criteria are generally grouped by body system.
- Some protocols may have additional protocol specific grading criteria, which will supersede the use of these tables for specified criteria

**LOCAL REACTIONS**				
	**GRADE 1**	**GRADE 2**	**GRADE 3**	**GRADE 4**
Induration	< 10mm	10-25 mm	26-50mm	>50mm

Erythema	< 10mm	10-25 mm	26-50mm	>50mm
Edema	< 10mm	10-25 mm	26-50mm	>50mm
Rash at Injection Site	< 10mm	10-25 mm	26-50mm	>50mm
Pruritus	Slight itching at injection site	Moderate itching at injection extremity	Itching at injection extremity and other sites	Itching over entire body
**GASTROINTESTINAL**				
	**GRADE 1**	**GRADE 2**	**GRADE 3**	**GRADE 4**
AST (SGOT)	1.1 - <2.0 x ULN	2.0 – <3.0 x ULN	3.0 – 8.0 x ULN	> 8 x ULN
ALT (SGPT)	1.1 - <2.0 x ULN	2.0 – <3.0 x ULN	3.0 – 8.0 x ULN	> 8 x ULN
GGT	1.1 - <2.0 x ULN	2.0 – <3.0 x ULN	3.0 – 8.0 x ULN	> 8 x ULN
**HEMATOLOGY**				
	**GRADE 1**	**GRADE 2**	**GRADE 3**	**GRADE 4**
Absolute Neutrophil Count	750-1200/mm^3	400-749/mm^3	250-399/mm^3	<250/mm^3

**ELECTROLYTES**				
	**GRADE 1**	**GRADE 2**	**GRADE 3**	**GRADE 4**
Hypernatremia		145-149 mEq/L	150-155 mEq/L	>155 mEq/L or abnormal sodium
Hyperkalemia	5.0-5.9 mEq/L	6.0-6.4 mEq/L	6.5-7.0 mEq/L	>7.0 mEq/L or abnormal potassium AND cardiac arrhythmia

Hypokalemia	3.0-3-5 mEq/L	2.5-2.9 mEq/L	2.0-2.4 mEq/L	<2.0 mEq/L or abnormal potassium AND cardiac arrhythmia
Hyperglycemia	116-159 mg/dL	160-249 mg/dL	250-400 mg/dL	>400 mg/dL or ketoacidosis

FOR LABORATORY PARAMETERS IMPACTED BY CEREBRAL MALARIA WHICH ARE EXPECTED TO BE DISORDERED AT BASELINE (PRIOR TO LVT ADMINISTRATION)

For evaluation at 24 hours post LVT Administration

	GRADE 1	GRADE 2	GRADE 3	GRADE 4
Bilirubin	No improvement from baseline	20% increase from baseline	30% increase from baseline	50% increase from baseline
Hemoglobin	Not applicable as ongoing anemia and need for blood transfusions not unexpected.			
Platelets	No improvement from baseline	20% decrease from baseline	30% decrease from baseline	50% decrease from baseline
Serum sodium	No improvement from baseline	20% decrease from baseline	30% decrease from baseline	50% decrease from baseline
Serum glucose	Not applicable as hypoglycemia during first 72 hours after admission with CM is not unexpected and is closely monitored for.			

For evaluation at 7 days post LVT Administration

	GRADE 1	GRADE 2	GRADE 3	GRADE 4
Bilirubin	No improvement from 24 hours post LVT	20% increase from 24 hours post LVT	30% increase from 24 hours post LVT	50% increase from 24 hours post LVT
Hemoglobin	No improvement from baseline but increased reticulocytes	No improvement from baseline and no increase in reticulocytes	Decreased from last measured but increased reticulocytes	Decreased from last measured and no increase in reticulocytes
Platelets	-------------------	50,000-75,000/mm^3	25,000-49,999/mm^3	<25,000/mm^3
Hypernatremia		145-149 mEq/L	150-155 mEq/L	>155 mEq/L or abnormal sodium
Hyponatremia		130-135 mEq/L	129-124 mEq/L	<124 mEq/L or abnormal sodium

OTHER				
	**GRADE 1**	**GRADE 2**	**GRADE 3**	**GRADE 4**
Allergy	Pruritus without Rash	Pruritic Rash	Mild Urticaria	Severe Urticaria Anaphylaxis, Angioedema
Cutaneous	Localized rash	Diffuse maculopapular Rash	Generalized urticaria	Stevens-Johnson Syndrome or Erythema multiforme
Stomatitis	Mild discomfort	Painful, difficulty swallowing, but able to eat and drink	Painful: unable to swallow solids	Painful: unable to swallow liquids; requires IV fluids
Clinical symptoms *not otherwise specified* in this table	No therapy; monitor condition	May require minimal intervention and monitoring	Requires medical care and possible hospitalization	Requires active medical intervention, hospitalization, or hospice care
Laboratory values *not otherwise specified* in this table	Abnormal, but requiring no immediate intervention; follow	Sufficiently abnormal to require evaluation as to causality and perhaps mild therapeutic intervention, but not of sufficient severity to warrant immediate changes in study drug	Sufficiently severe to require evaluation and treatment, including at least temporary suspension of study drug	Life-threatening severity; Requires immediate evaluation, treatment, and usually hospitalization; Study drug must be stopped immediately and should not be restarted until the abnormality is clearly felt to be caused by some other mechanism that study drug
