# Supplementary material for: A clinical trial of enteral Levetiracetam for acute seizures in pediatric cerebral malaria
Source: BMC Pediatr. 2019 Nov 1;19:399. doi: 10.1186/s12887-019-1766-2 (PMC6824014; doi:10.1186/s12887-019-1766-2)
Supplement: Supplementary file 3 — Additional file 3. Q-Q Plots for Evaluation of Normalcy [file 12887_2019_1766_MOESM3_ESM.pdf]

Date: September 22, 2019

Supplementary Materials

Q-Q Plots for Evaluation of Normalcy

For continuous variables in Tables 3, quantile-quantile (QQ) plots were obtained in the (independent) LVT and PB groups. Log- transformation was applied to mitigate skewness in some variables. Comparisons between groups were made by t-tests or Mann-Whitney-Wilcoxon U-tests (MWW) as appropriate.

- **AGE in months (Age_mts)**

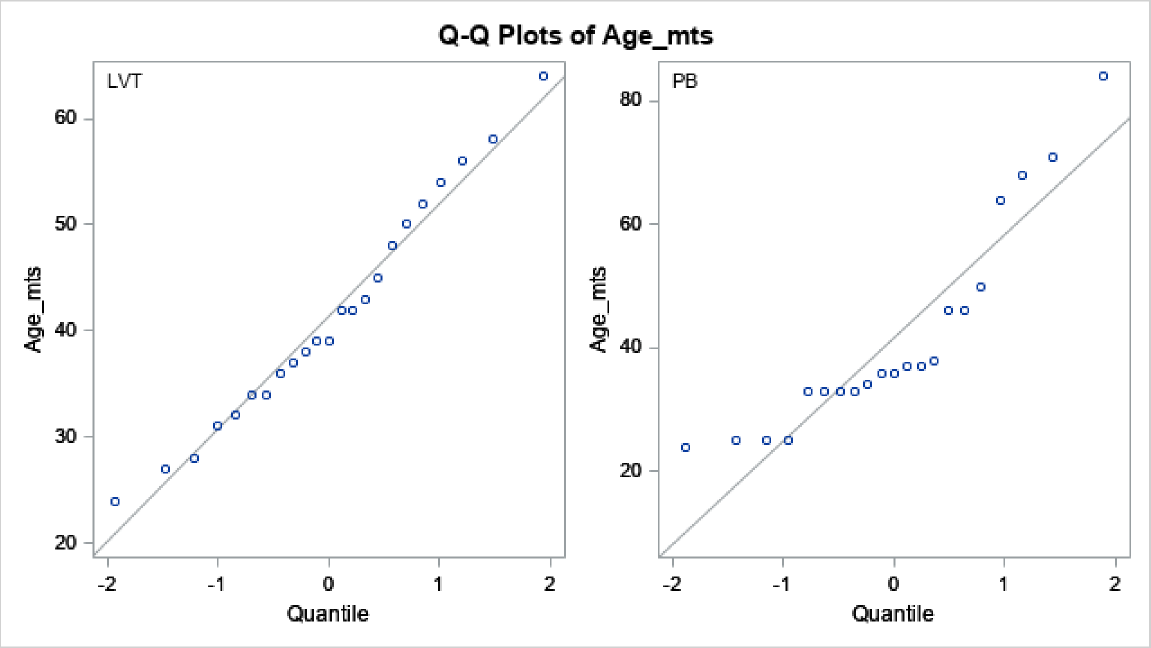

In LVT the normality assumption is tenable, but not so in PB.

Comparison based on MWW test: p=0.50

Date: September 22, 2019

- **Parasite count (qmp)**

Parasite count was log-transformed [L_qmp] and then compared between LVT and PB

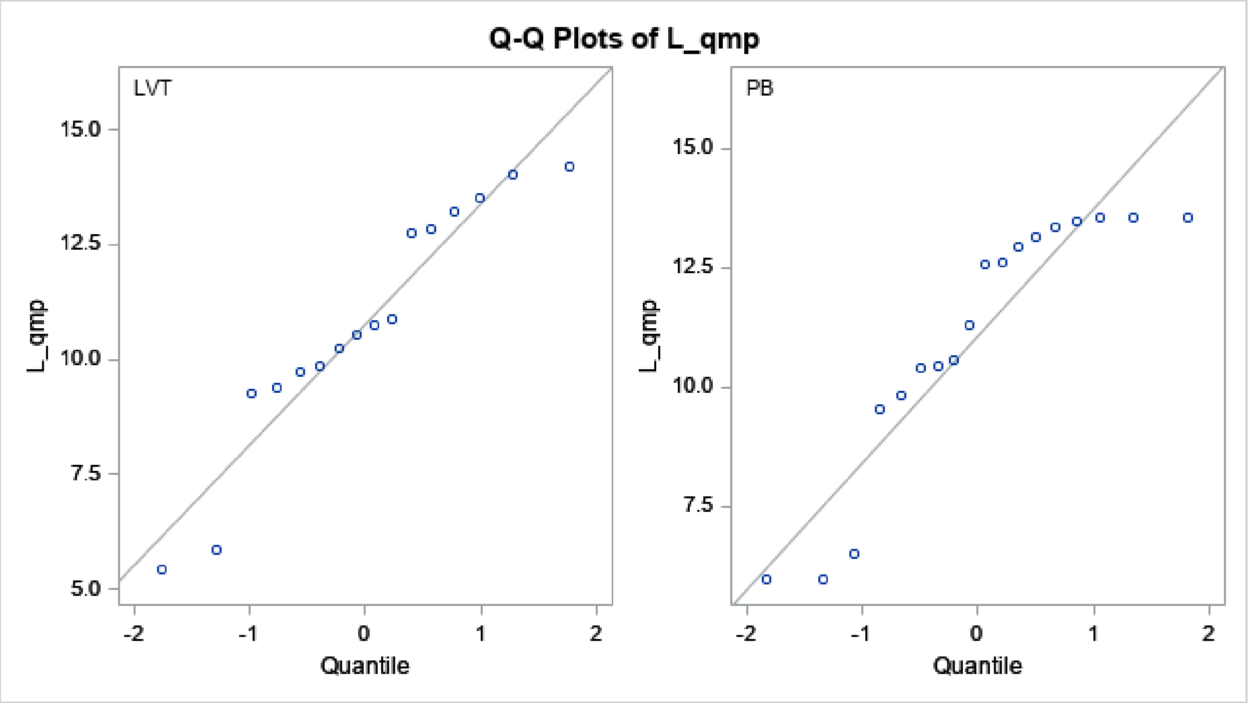

Normality tenable in LVT, but not in PB

Comparison based on MWW test: p=0.62 for positive parasite counts, and separately for proportion of zero counts. Zero Counts: In LVT 7/23 (30%); in PB 3/21 (14%). Difference not significant (p=0.29).

- **Lactate**

Log-transformation applied to reduce skewness before comparison is made

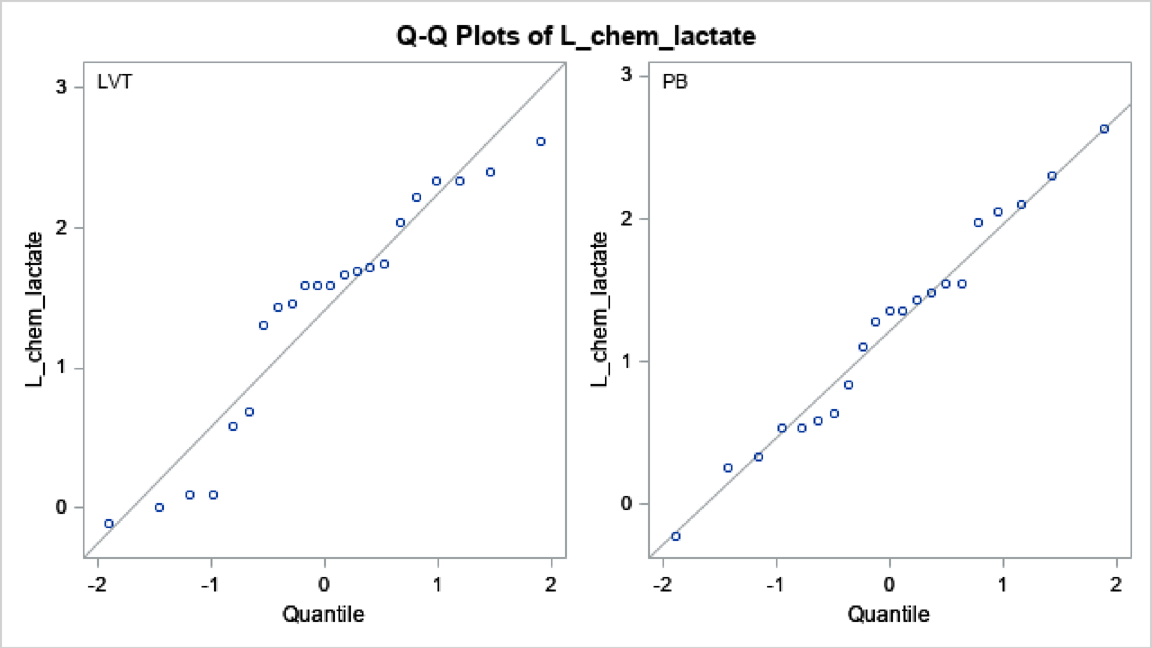

Comparison based on t-test: p=0.43 on log transformed values.

- **Hemoglobin (HB)**

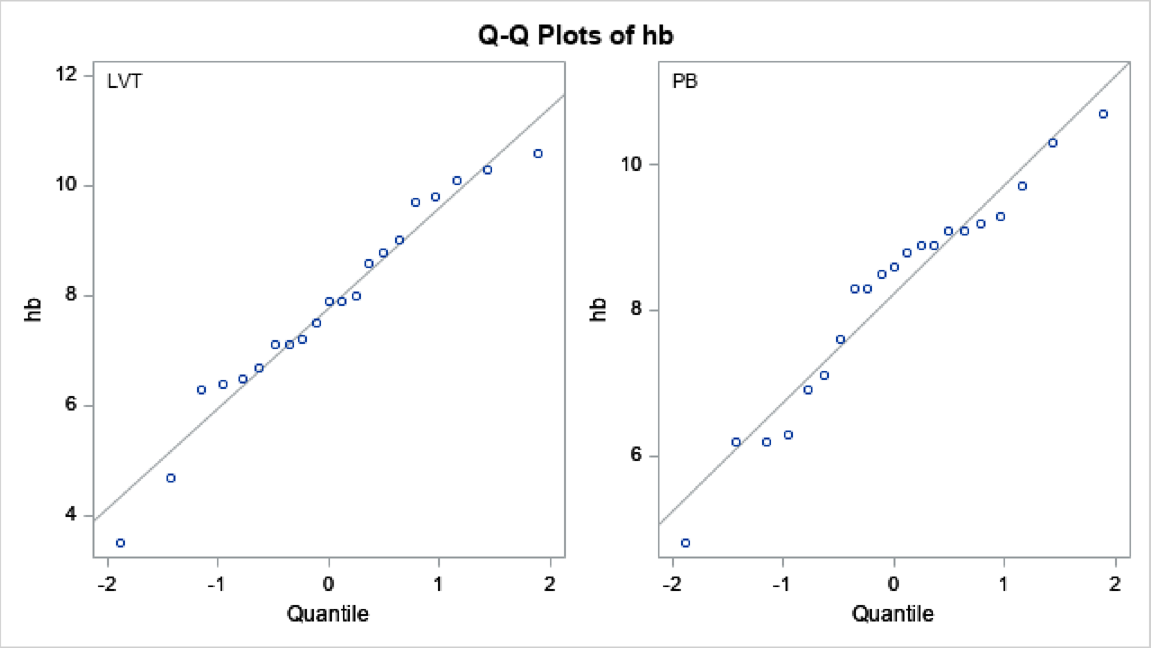

In LVT one outlier was removed. Normality tenable in both groups.

Comparison based on t-test: p=0.41

- **Hematocrit (hct)**

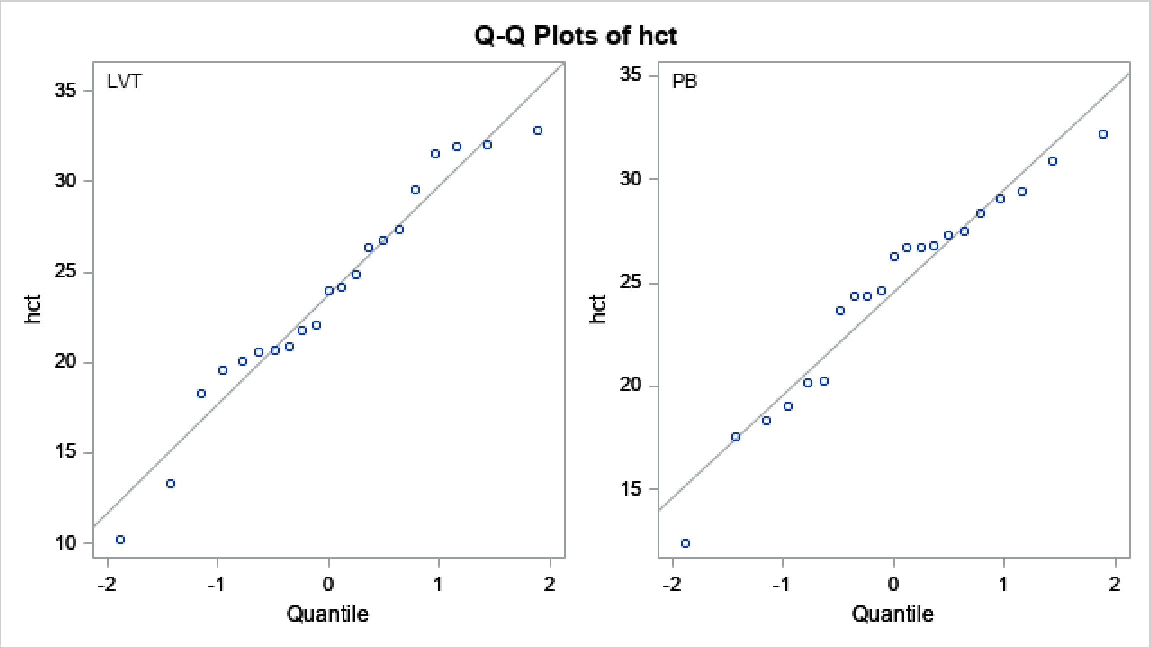

In LVT one outlier was removed. Normal distribution is tenable in both groups

Comparison based on t-test: p=0.63

- **Platelets**

The log-transformation was applied to mitigate skewness.

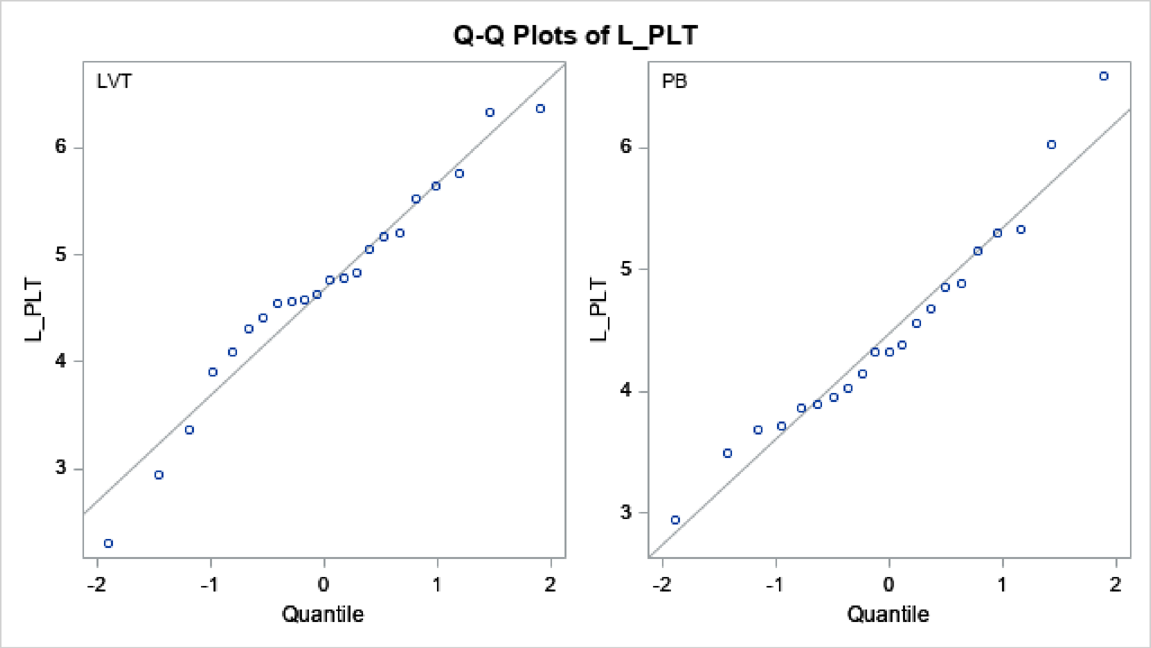

Normality tenable in each group on log transformed values.

Comparison by t-test: p=0.48
