# Supplementary material for: A clinical trial of enteral Levetiracetam for acute seizures in pediatric cerebral malaria
Source: BMC Pediatr. 2019 Nov 1;19:399. doi: 10.1186/s12887-019-1766-2 (PMC6824014; doi:10.1186/s12887-019-1766-2)
Supplement: Supplementary file 4 — Additional file 4. Supplementary Table 1: Neurologic Sequelae [file 12887_2019_1766_MOESM4_ESM.pdf]

Supplementary TABLE: Neurologic Sequelae

| Allocation | Sequelae |
|---|---|
| LVT | LVT008:Cortically blind, deaf and severely hypotonic<br><br>LVT009: Hemiparesis and ataxia with titubation<br><br>LVT050: Deaf, blind and with abnormal movements |
| PB | LVT037: Ataxia and hemiparesis<br><br>LVT047: Hypotonic and blind |
