# Supplementary material for: A clinical trial of enteral Levetiracetam for acute seizures in pediatric cerebral malaria
Source: BMC Pediatr. 2019 Nov 1;19:399. doi: 10.1186/s12887-019-1766-2 (PMC6824014; doi:10.1186/s12887-019-1766-2)
Supplement: Supplementary file 5 — Additional file 5. Supplementary Table 2: Serious Events by Allocation [file 12887_2019_1766_MOESM5_ESM.pdf]

Supplementary TABLE: Serious Adverse Events (SAE) by Allocation*

	**Enteral Levetiracetam (n=23)**	**Usual Care (n=21) Phenobarbital Intent-to-Treat**	**Phenobarbital received per protocol (n=15)**
Any SAE	5 (22%)	8 (38%)	8 (53%)
Thrombocytopenia^	2 (9%)	1 (5%)	1 (7%)
Elevated AST	3 (13%)	2 (10%)	2 (13%)
Acute kidney injury	1 (4%)	0	0
**Respiratory suppression/aspiration**	**0**	**4 (19%)**	**4 (27%)**
Death	1 (4%)	5 (24%)	5 (33%)

* All comparisons p>0.05. SADRs are noted in **bold**

^ 50% decrease from baseline at 24 hours post randomization or <25,000 per µL at 7 days post randomization
