# Supplementary material for: A clinical trial of enteral Levetiracetam for acute seizures in pediatric cerebral malaria
Source: BMC Pediatr. 2019 Nov 1;19:399. doi: 10.1186/s12887-019-1766-2 (PMC6824014; doi:10.1186/s12887-019-1766-2)
Supplement: Supplementary file 6 — Additional file 6. Supplementary Table 3: Adverse Events, not Serious [file 12887_2019_1766_MOESM6_ESM.pdf]

Supplementary TABLE: Adverse Events, not Serious

	**Enteral Levetiracetam (n=23)**	**Usual Care (n=21) (+/- Phenobarbital)**
Any adverse event, not serious	15 (65%)	18 (86%)
Anemia^	0	1 (5%)
Thrombocytopenia*	2 (9%)	1 (5%)
Thrombocytosis	2 (9%)	1 (5%)
Decreased reticulocyte count	1 (4%)	4 (19%)
Abnormal ECG	0	1 (5%)
Vomiting	1 (4%)	0
Increased AST	5 (22%)	7 (33%)
Increased ALT	4 (17%)	4 (19%)
Increased alkaline phosphatase	3 (13%)	5 (24%)
Increased potasssium	7 (31%)	4 (19%)
Increased chloride	2 (9%)	0
Increased phosphate	6 (26%)	1 (5%)
Increased calcium	1 (4%)	0
**Myoclonus (transient)**	**1 (4%)**	**0**
**Persistent somnolence**	**0**	**1 (5%)**

^ Hemoglobin at 7 days post randomization decreased compared to hemoglobin at 24 hours post randomization without associated increase in reticulocytes

* 50% decrease from baseline at 24 hours post randomization or <25,000 per µL at 7 days post randomization

SADRs are noted in **bold**
